# Orchard: Building large cancer phylogenies using stochastic combinatorial search

**DOI:** 10.1371/journal.pcbi.1012653

**Published:** 2024-12-30

**Authors:** Ethan Kulman, Rui Kuang, Quaid Morris

**Affiliations:** 1 Computational and Systems Biology Program, Memorial Sloan Kettering Cancer Center, New York, New York, United States of America; 2 Department of Computer Science and Engineering, University of Minnesota, Minneapolis, Minnesota, United States of America; University of Illinois at Urbana-Champaign, UNITED STATES OF AMERICA

## Abstract

Phylogenies depicting the evolutionary history of genetically heterogeneous subpopulations of cells from the same cancer, i.e., *cancer phylogenies*, offer valuable insights about cancer development and guide treatment strategies. Many methods exist that reconstruct cancer phylogenies using point mutations detected with bulk DNA sequencing. However, these methods become inaccurate when reconstructing phylogenies with more than 30 mutations, or, in some cases, fail to recover a phylogeny altogether. Here, we introduce *Orchard*, a cancer phylogeny reconstruction algorithm that is fast and accurate using up to 1000 mutations. Orchard samples without replacement from a factorized approximation of the posterior distribution over phylogenies, a novel result derived in this paper. Each factor in this approximate posterior corresponds to a conditional distribution for adding a new mutation to a partially built phylogeny. Orchard optimizes each factor sequentially, generating a sequence of incrementally larger phylogenies that ultimately culminate in a complete tree containing all mutations. Our evaluations demonstrate that Orchard outperforms state-of-the-art cancer phylogeny reconstruction methods in reconstructing more plausible phylogenies across 90 simulated cancers and 14 B-progenitor acute lymphoblastic leukemias (B-ALLs). Remarkably, Orchard accurately reconstructs cancer phylogenies using up to 1,000 mutations. Additionally, we demonstrate that the large and accurate phylogenies reconstructed by Orchard are useful for identifying patterns of somatic mutations and genetic variations among distinct cancer cell subpopulations.

## Author summary

Somatic mutations accumulate during cancer progression, leading to genetically distinct subpopulations of cancer cells. Understanding the evolutionary relationships among these subpopulations reveals critical events and offers clinical insights for treatment. We developed a novel machine learning algorithm, Orchard, which uses point mutations detected via bulk DNA sequencing to reconstruct phylogenies depicting the genetic evolution of an individual cancer. We evaluated Orchard on 90 simulated cancers and 14 B-progenitor acute lymphoblastic leukemias. Our results demonstrate that Orchard is fast and accurate at reconstructing phylogenies with up to 1,000 mutations detected in 100 cancer samples. Orchard’s ability to reconstruct large phylogenies motivated a novel approach: applying agglomerative clustering to these trees to identify clusters of mutations that co-occur in cells. Our results show that this new clustering approach accurately identifies mutation clusters in real data, often outperforming state-of-the-art methods.

## 1 Introduction

Cancerous cells contain somatic mutations that override normal cellular controls [1, 2]. Evolution progresses as cancer cells acquire additional mutations that provide selection advantages [3], leading to genetically distinct cancerous cell subpopulations (i.e., *subclones*) characterized by sets of shared mutations [4]. Phylogenies describing the ancestral relationships of subclones in an individual cancer can reveal important evolutionary events, help to characterize the cancer’s heterogeneity [5] and progression [6, 7], support the discovery of new driver mutations [8], and provide clinical insight for treatment regimens [9]. Many cancer evolution studies use bulk DNA from multiple cancer samples to reconstruct phylogenies depicting the evolution of subclones in an individual cancer [10–16]. These phylogenies are also prerequisites for inferring migration histories of metastases [17, 18].

A *mutation tree* is an arborescence that can represent portions of a cancer phylogeny that satisfy the infinite sites assumption (ISA). The ISA posits that each mutation is acquired only once and is never reverted. A phylogeny that adheres to the ISA is a *perfect phylogeny*; cancer phylogenies are often perfect because of low mutation rates (∼1–10 mutations / megabase) [19]. By definition, a mutation tree is a perfect phylogeny, and each non-root node in a mutation tree represents a somatic mutation present in one or more cancerous cell subpopulations. Bulk DNA data obtained by sequencing multiple tissue samples from the same cancer can be used to reconstruct a mutation tree by solving the *mixed sample perfect phylogeny* (MSPP) problem.

In the MSPP problem, each cancer sample is an unknown mixture of clonal genotypes, i.e, *clones*, that are related to one another via a perfect phylogeny. The input to the MSPP problem is a matrix of the frequencies of each mutation in each sample; these frequencies can be estimated from the variant allele frequencies (VAFs) measured by short read sequencing of the bulk samples. Although perfect phylogeny reconstruction has a linear time algorithm [20], MSPP reconstruction is NP-complete even if the mutation frequency estimates are noise-free [21]. There are some special cases where the noise-free MSPP problem can be solved in quadratic time [22–24].

Until recently, no MSPP reconstruction algorithm could reliably build mutation trees with more than 10 mutations [25]. State-of-the-art MSPP reconstruction algorithms such as Pairtree [25] and CALDER [26] are capable of accurately reconstructing 30-node mutation trees, and in some rare cases, 100-node mutation trees. However, most MSPP problems have more than 30 mutations, and this necessitates that mutations are pre-clustered based on their VAFs prior to MSPP reconstruction [27–29]. When mutations are clustered together, the reconstructed tree is known as a *clone tree*, and many MSPP algorithms treat each pre-defined cluster of mutations as a single mutation during reconstruction. Clustering mutations is often referred to as subclonal reconstruction, because these mutation sets uniquely define *subclones* which are subpopulations of cancer cells that correspond to clades in the phylogeny. Unfortunately, using mutation VAFs to define subclones is an error-prone process that can lead to incorrectly estimating the number of subclones [30, 31], and incorrectly grouping mutations together that are in unrelated clones [32]. Reconstructing a clone tree using poorly defined subclones can yield inaccurate trees and lead to incorrect conclusions about the evolution of a cancer [31–33]. Pre-defining subclones before tree reconstruction can also result in the loss of critical information such as the partial order of mutation acquisition, which is crucial for analyzing somatic mutation patterns and determining prognosis and treatment strategies for certain hematological cancers [6, 7, 34–36].

Here, we introduce a new MSPP reconstruction algorithm, Orchard, which reliably reconstructs extremely large mutation trees, alleviating the need to pre-cluster mutations. Orchard accurately solves MSPP problems with as many as 1000 mutations and 100 samples. We benchmark Orchard’s reconstructions against Pairtree and CALDER on 14 B-progenitor acute lymphoblastic leukemias (B-ALLs) and 90 simulated cancers. These results demonstrate that: (1) Orchard matches or outperforms Pairtree and CALDER on reconstruction problems with 30 or fewer mutations while being 5–10x faster, and (2) Orchard greatly outperforms both competing methods on reconstruction problems with more than 30 mutations. Orchard achieves greater accuracy and scalability for large reconstruction problems by sampling from a factorized approximation of the mutation tree posterior, a novel result derived in this paper. Each factor in this approximate posterior represents a conditional distribution for adding a new mutation to a partially built tree. Consequently, Orchard samples from this approximate posterior by generating sequences of progressively larger trees, culminating in a complete tree containing all mutations. Orchard uses Gumbel Soft-Max Tricks [37–39] to sample trees for each factor, enabling it to sample complete mutation trees without replacement. This approach differs from existing methods, such as Pairtree [25], which uses Markov Chain Monte Carlo (MCMC) to explore the space of complete mutation trees, or CALDER [26], which formulates the MSPP problem as a mixed integer linear program (MILP). These existing algorithmic approaches have critical pitfalls: MCMC-based methods often converge slowly and are prone to becoming stuck in local maxima because samples are highly auto-correlated, while MILP-based methods can be extremely slow, may fail to recover a solution, or may only be able to produce a single solution in a reasonable amount of time. Orchard avoids these pitfalls because of two crucial properties of the algorithm: it samples without replacement, which mitigates the risk of becoming stuck in local maxima, and its search strategy retains and grows partial tree structures that adhere closely to the ISA, effectively pruning large portions of the search space.

Orchard’s ability to accurately reconstruct large mutation trees enables a novel strategy to flexibly define subclones. To illustrate this new strategy, we introduce a simple *phylogeny-aware* clustering algorithm that infers subclones from a reconstructed mutation tree. On real data, this simple algorithm recovers expert-defined subclones as well as, and often better, than state-of-the-art VAF based clustering algorithms [27–29]. Orchard, along with the phylogeny-aware clustering algorithm, is accessible for free under the MIT License at http://www.github.com/morrislab/orchard.

## 2 Materials and methods

Orchard reconstructs cancer phylogenies using point mutations detected via bulk DNA sequencing. Below, we first describe the mixed sample perfect phylogeny (MSPP) problem and its definition using variant allele frequency data. Next, we derive a novel factorized approximation to the mutation tree posterior and demonstrate how to use Gumbel Soft-Max Tricks [37, 38] to sample without replacement from it. We then present the Orchard algorithm’s pseudo-code and discuss implementation-specific details. Lastly, we describe our phylogeny-aware clustering algorithm that infers clones and clone trees using a mutation tree.

### 2.1 The cancer-specific mixed sample perfect phylogeny problem

The input to a mixed sample perfect phylogeny problem is a matrix of *mutation frequencies*, $F\in R^{n\times m}$, and the goal is to infer a perfect-phylogeny-compatible binary genotype matrix *B* ∈ {0, 1}_*n*×*n*_. Each column *B*_:*v*_ in *B* represents the binary genotype for clone *v*. The rows of *B* represent mutations, where if *B*_*jv*_ = 1, then clone *v*’s genome contains mutation *j*. The rows of *F* represent mutations and the columns represent samples. Each entry *F*_*js*_ is the frequency of mutation *j* in sample *s*, where *F*_*js*_ ∈ [0, 1]. Since multiple clones may harbor mutation *j*, *F*_*js*_ equals the sum of the frequency of those clones in sample *s*, i.e., *F*_*js*_ = ∑_*v*_ *B*_*jv*_*U*_*vs*_, where *U*_*vs*_ ≥ 0 and ∑_*v*_*U*_*vs*_ ≤ 1. The mixing coefficients, *U*_*vs*_, represent the proportion of cells in samples *s* with the clonal genotype *v*, i.e., the *clonal proportions*. The general relationship between *F*, *B*, and *U*, is:

$$\begin{array}{c} F=BU. \end{array}$$ (1)

The basic MSPP problem assumes that *F* is noise free, and solving it requires finding a perfect-phylogeny-compatible binary genotype matrix *B* and a *clonal proportion matrix* $U\in R^{n\times m}$ that satisfy Eq 1.

In the cancer-specific, noisy MSPP problem, we assume that *F* cannot be observed directly, and instead, we are provided variant allele frequency data *D*, from which a noisy estimate $\hat{F}$ of *F* can be derived. The goal is to find *B* and *U* that admit an *F* with the highest possible data fit to *D*. The next section describes how we define a noisy MSPP problem, one step of which is estimating $\hat{F}$ using the observed bulk DNA data. After that, we formally define a mutation tree.

### 2.2 The input to Orchard

The inputs to Orchard include VAFs for *n* point mutations in *m* samples from the same cancer. For each point mutation *j*, Orchard requires a set of tuples $X_{j}=\{(a_{js},b_{js},\omega_{js}){\}}_{s=1}^{m}$, where *s* indexes the bulk cancer sample. The entire data set for all *n* mutations is represented by a set $D=\{X_{j}{\}}_{j=1}^{n}$.

Each tuple (*a*_*js*_, *b*_*js*_, *ω*_*js*_) ∈ *X*_*j*_ represents the bulk DNA data from sample *s* for the genomic locus containing mutation *j*: *b*_*js*_ is the count of reads containing the variant allele *j*; *a*_*js*_ is the count of reads containing the reference allele; and *ω*_*js*_ is the *variant read probability*, which is the estimated proportion of alleles per cancer cell that contain the variant allele *j*. The latter value, 0 ≤ *ω*_*js*_ ≤ 1, is required to convert from VAFs to mutation frequencies; and the dependence on *s* permits modelling of sample-specific copy number aberrations (CNAs). Under the ISA, a mutation *j* initially only affects one allele, so in diploid and haploid regions free of CNAs, $\omega_{js}=\frac{1}{2}$ and *ω*_*js*_ = 1, respectively. In some cases, *ω*_*js*_ can be estimated by performing an allele-specific copy number analysis [40]. If mutations are heavily impacted by CNAs, Orchard requires reliable sample specific copy number estimates to produce accurate reconstructions; if these are unavailable, mutations affected by CNAs should be omitted from the inputs provided to Orchard.

The observed VAF for mutation *j* in sample *s*, denoted by ${\hat{\lambda }}_{js}$, is given by ${\hat{\lambda }}_{js}=\frac{b_{js}}{b_{js}+a_{js}}$. This VAF can be converted to an observed mutation frequency, ${\hat{F}}_{js}$, using the following formula:

$$\begin{array}{c} {\hat{F}}_{js}={\hat{\lambda }}_{js}/\omega_{js}. \end{array}$$ (2)

The value of ${\hat{F}}_{js}$ is an estimate of the percentage of cells in sample *s* that have mutation *j*.

### 2.3 Mutation tree representations of perfect phylogenies

Orchard represents perfect phylogenies of cancer clones using a mutation tree. A mutation tree is a perfect phylogeny represented as a rooted, directed tree, i.e., an arborescence. We denote it with *t* = {*V*, *E*, *M*}, where *V* is a set of nodes, indexed by *v* ∈ {*r*} ∪ {1, 2, …, *n*}, with *r* indicating the root node representing the germline; *E* is a set of directed edges defining the child/parent relationships among the nodes; and *M* is a vector of mutations, one for each non-root node. Each node *v* ∈ *V* \ {*r*} defines a genetically distinct cell population or *clone* whose clone-defining mutation is *M*_*v*_. Each directed edge (*v*, *u*) ∈ *E* indicates that *v* is a parent of *u*, and that *u* inherits the mutation *M*_*v*_, along with all mutations that *v* inherited from its ancestors. One implication of this inheritance is that the set of clones in the subtree rooted at *v* are uniquely defined by having the mutation *M*_*v*_, thus *M*_*v*_ also defines a subclone rooted at *v*. For convenience, we will assign the same index to a clone, its clone-defining mutation, and the node associated with the clone, so clone *v* is represented by mutation *v* and *M*_*v*_ = *v*.

Orchard also considers *partial mutation trees*, denoted as *t*^(*ℓ*)^ = {*V*^(*ℓ*)^, *E*^(*ℓ*)^, *M*^(*ℓ*)^}, where the superscript *ℓ* denotes the number of mutations the tree contains. A tree containing only the root node and a single mutation is denoted as *t*^(1)^, and a *complete mutation tree* containing all *n* mutations and the root node is denoted as *t*^(*n*)^. If 0 < *ℓ* < *n*, then *V*^(*ℓ*)^ ⊂ *V* and only the entries *M*_*v*_ for *v* ∈ *V*^(*ℓ*)^ \ {*r*} are defined. To complete a partial tree *t*^(*ℓ*)^, the remaining mutations *V* \ *V*^(*ℓ*)^ need to be added to *t*^(*ℓ*)^. Adding *v* ∈ *V* \ *V*^(*ℓ*)^ to *t*^(*ℓ*)^ results in a new tree *t*^(*ℓ*+1)^ = {*V*^(*ℓ*)^ ∪ {*v*}, *E*^(*ℓ*+1)^, *M*^(*ℓ*+1)^}. The mutation *v* can be incorporated into *t*^(*ℓ*)^ either as an internal node, where it is ancestral to one or more mutations already in *t*^(*ℓ*)^, or as a leaf node.

**Fig 1:**
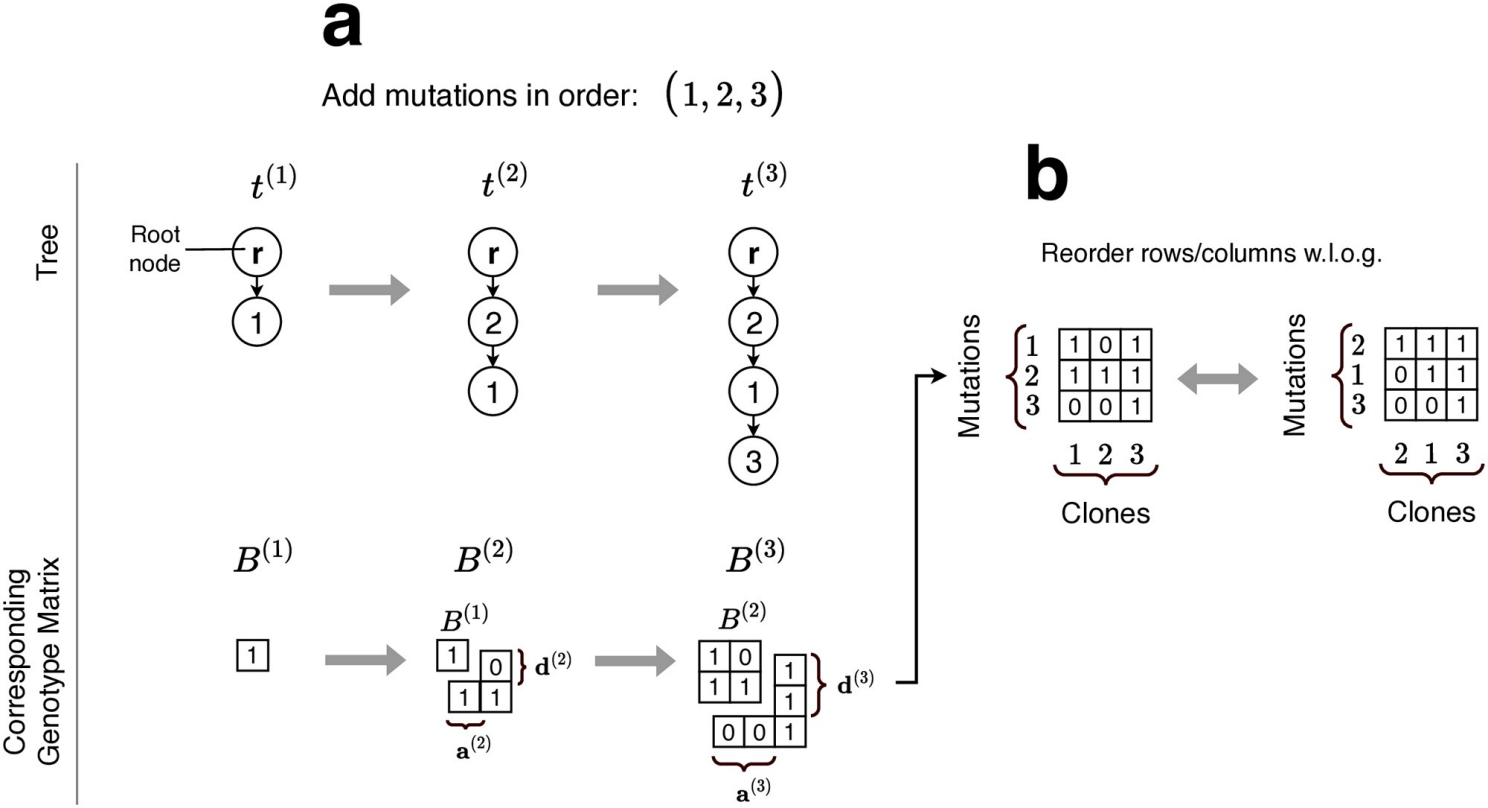
Overview of how adding a new mutation changes a tree and its binary genotype matrix. **a**. Introducing a new mutation to the tree *t*^(*ℓ*)^ corresponds to adding a new row **a**^(*ℓ*+1)^ and column **d**^(*ℓ*+1)^ to *B*^(*ℓ*)^. These additions must be filled in such a way that *B*^(*ℓ*+1)^ remains a perfect-phylogeny-compatible binary genotype matrix. **b**. The rows and columns of a binary genotype matrix can be reordered without affecting the underlying tree structure. If we reorder the rows and columns such that ancestors come before their descendants, then the matrix becomes upper triangular as shown here.

Each unique mutation tree is consistent with at most one binary genotype matrix *B* that can be resolved in linear time [20], and vice versa. Consequently, we establish equivalent representations for binary genotype matrices as *t*^(1)^ ≡ *B*^(1)^, *t*^(*ℓ*)^ ≡ *B*^(*ℓ*)^, and *t*^(*n*)^ ≡ *B*^(*n*)^. Adding *v* ∈ *V* \ *V*^(*ℓ*)^ to *t*^(*ℓ*)^ uniquely extends its binary genotype matrix *B*^(*ℓ*)^. If *t*^(*ℓ*+1)^ is defined as above, then $B^{\left(\ell +1\right)}=[\begin{array}{c} B^{\left(\ell \right)},{\text{a}}^{\left(\ell +1\right)};{\text{d}}^{\left(\ell +1\right)},1 \end{array}]$, where **a**^(*ℓ*+1)^ is a length *ℓ* binary column vector indicating *v*’s ancestors in *t*^(*ℓ*+1)^, **d**^(*ℓ*+1)^ is a length *ℓ* binary row vector indicating *v*’s descendants, and *B*^(*ℓ*)^ is unchanged. Without loss of generality, we can re-order the rows and columns of *B*, and its submatrices *B*^(*ℓ*)^, so that if the *v*-th row of *B*^(*ℓ*)^ represents the genotype of clone *v*, then the *v*-th column of *B*^(*ℓ*)^ corresponds to the clone defining mutation *M*_*v*_ for clone *v*. As such $B_{v,v}^{\left(\ell \right)}=1$ for any *v*. This row and column ordering guarantees that if $B_{v,w}^{\left(\ell \right)}=1$, then either *v* = *w* or clone *v* is an ancestor of clone *w* in *t*^(*ℓ*)^. Fig 1 depicts the relationship between *t*^(*ℓ*)^ and *B*^(*ℓ*)^ as mutations are added, and demonstrates how the rows and columns of the genotype matrix can be reordered without affecting the underlying tree structure.

### 2.4 Motivating the approximate posterior

Orchard’s goal is to sample without replacement from the mutation tree posterior defined by:

$$\begin{array}{c} P\left(B|D\right)=\frac{P(B,D)}{P(D)}, \end{array}$$ (3)

where *B* is a genotype matrix and *D* is a data set for *n* point mutations across *m* bulk samples. Note that computing *P*(*B*, *D*) = ∫_*U*_*P*(*B*, *U*, *D*)*dU* requires marginalizing over the clonal proportion matrix *U*. According to Cayley’s formula [41], (*n* + 1)^*n*−1^ unique mutation trees can be constructed with *n* mutations and a root node *r*. Consequently, if *D* contains data for a large number of mutations, then it is intractable to compute the normalizing constant, *P*(*D*) = ∑_*B*_ ∫_*U*_ *P*(*B*, *U*, *D*)*dU*, since this requires instantiating (*n* + 1)^*n*−1^ perfect-phylogeny-compatible *B*-matrices [41], and integrating over the possible values of *U* for each *B*. Fortunately, there are a variety of Markov Chain Monte Carlo (MCMC) techniques which do not require computing *P*(*D*). These techniques rely on the following:

$$\begin{array}{cc} P(B|D) & \propto P(B,D)\\ & \propto \int_{U}P\left(B,U,D\right)dU\\ & \propto \int_{U}P\left(D|B,U\right)P\left(U|B\right)P\left(B\right)dU. \end{array}$$ (4)

Eq 4 can be used to design an MCMC sampling algorithm, where *B*-matrices are sampled and subsequently evaluated using the likelihood *P*(*D*|*B*, *U*) by computing (or estimating) the integral over *U*, see, e.g., [25]. MCMC methods can be slow to converge and mix, and if *P*(*B*|*D*) has low entropy, then many samples will need to be drawn before *k* distinct ones are obtained. Here, we propose an approximation to the posterior, *Q*^*π*^(*B*|*D*), from which one can sample trees containing all *n* mutations by progressively adding one mutation at a time to a partial tree structure. We will then show how Gumbel-Max tricks [37–39] can be used to efficiently sample without replacement from *Q*^*π*^(*B*|*D*).

We define *Q*^*π*^(*B*|*D*) by choosing a fixed order in which mutations are added to the tree; the accuracy of its approximation of *P*(*B*|*D*) depends on this ordering. We denote a mutation order with the vector **π** representing a permutation, whose *ℓ*-th element, *π*_*ℓ*_, is the index of the *ℓ*-th mutation added. Each mutation appears in *π* exactly once. We use the notation *D*^(*ℓ*+1)^ to represent the data associated with the first *ℓ* + 1 mutations in *π*. Given *π*, we write:

$$\begin{array}{c} Q^{\pi }\left(B|D\right)=\prod_{\ell =1}^{n-1}P\left(B^{\left(\ell +1\right)}|D^{\left(\ell +1\right)},B^{\left(\ell \right)}\right) \end{array}$$ (5)

$$\begin{array}{c} \approx \prod_{\ell =1}^{n-1}P\left(B^{\left(\ell +1\right)}|D,B^{\left(\ell \right)}\right)=P\left(B|D\right) \end{array}$$ (6)

Here, the genotype matrix *B*^(*ℓ*+1)^ is obtained from *B*^(*ℓ*)^ by appending **a**^(*ℓ*+1)^, representing the ancestors of the new mutation in *t*^(*ℓ*+1)^, and **d**^(*ℓ*+1)^, representing its descendants (see Section 2.3). Eq 6 follows directly from factoring *P*(*B*|*D*) into sets of binary random variables, i.e., {(**a**^(2)^, **d**^(2)^), (**a**^(3)^, **d**^(3)^), …, (**a**^(*n*)^, **d**^(*n*)^)} associated with the elements in the newly added row and column in *B*^(*ℓ*+1)^ versus *B*^(*ℓ*)^ and noting that *B*^(1)^ is a 1 × 1 binary matrix containing a 1. See Section A3.2 in S1 Appendix for more details. Sampling from *Q*^*π*^(*B*|*D*) in place of *P*(*B*|*D*) is dramatically more efficient. Specifically, computing each factor *P*(*B*^(*ℓ*+1)^|*D*, *B*^(*ℓ*)^) requires evaluating all mutation trees of size *n* that include *B*^(*ℓ*+1)^ as a submatrix. In contrast, computing *Q*^*π*^(*B*^(*ℓ*+1)^|*D*^(*ℓ*+1)^, *B*^(*ℓ*)^) requires evaluating only mutation trees of size *ℓ* + 1 that contain *B*^(*ℓ*)^ as a submatrix. The difference in computational complexity is super-exponential.

In general, Eq 5 is equal to Eq 6 if and only if:

$$\begin{array}{c} B^{\left(\ell +1\right)}╨D\backslash D^{\left(\ell +1\right)}|B^{\left(\ell \right)},D^{\left(\ell +1\right)}, \end{array}$$ (7)

i.e., that, given *B*^(*ℓ*)^ and the data for the first *ℓ* + 1 mutations, *D*^(*ℓ*+1)^, *B*^(*ℓ*+1)^ is conditionally independent from the data for the last *n* − (*ℓ* + 1) mutations in *π*, *D* \ *D*^(*ℓ*+1)^. This conditional independence assumption states that data for mutations not yet in the tree can be ignored when placing new mutations. Ordering the mutations so that each mutation is placed in the tree before its descendants will often ensure that this approximation is accurate. However, there are counterexamples where this is not true. We discuss this issue in more detail in Section A2.1 in S1 Appendix.

Evaluating each placement of *π*_*ℓ*+1_ into *t*^(*ℓ*)^ requires computing the conditional likelihood *P*(*B*^(*ℓ*+1)^|*D*^(*ℓ*+1)^, *B*^(*ℓ*)^). This term can be rewritten as follows:

$$\begin{array}{c} P\left(B^{\left(\ell +1\right)}|D^{\left(\ell +1\right)},B^{\left(\ell \right)}\right)\propto P\left(D^{\left(\ell +1\right)}|B^{\left(\ell +1\right)}\right)P\left(B^{\left(\ell +1\right)}|B^{\left(\ell \right)}\right). \end{array}$$ (8)

We discuss computing the data likelihood *P*(*D*^(*ℓ*+1)^|*B*^(*ℓ*+1)^) in Section 2.5.5. Eq 8 introduces a new term, *P*(*B*^(*ℓ*+1)^|*B*^(*ℓ*)^), which we define as:

$$\begin{array}{c} P\left(B^{\left(\ell +1\right)}|B^{\left(\ell \right)}\right)=\{\begin{array}{cc} \tau^{-1} & \begin{array}{cc} & \text{if}\;B^{\left(\ell +1\right)}\;\text{is}\;\text{a}\;\text{perfect}\;\text{phylogeny}\;\text{genotype}\;\text{matrix}\;\text{and}\;\\ & B^{\left(\ell +1\right)}=[B^{\left(\ell \right)},{\text{a}}^{\left(\ell +1\right)};{\text{d}}^{\left(\ell +1\right)},1] \end{array}\\ 0 & \;\text{otherwise}, \end{array} \end{array}$$ (9)

where *τ* is the number of possible settings of **a**^(*ℓ*+1)^ and **d**^(*ℓ*+1)^ such that *B*^(*ℓ*+1)^ is a perfect phylogeny genotype matrix.

### 2.5 Orchard algorithm

**Fig 2:**
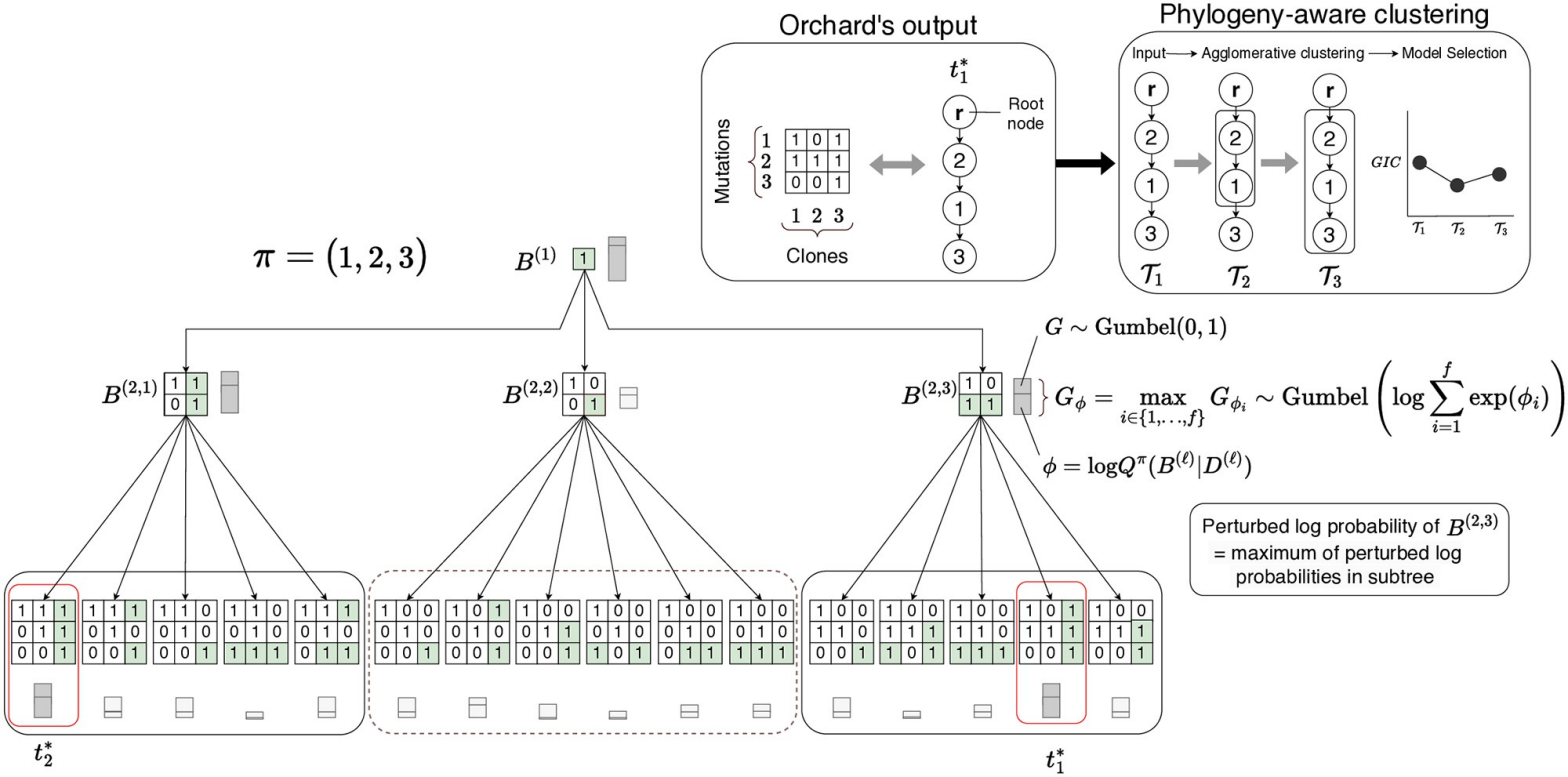
Example of Orchard’s mutation tree search with k = 2, *f* = ∞. Mutation trees are depicted using genotype matrices. Search begins with a genotype matrix *B*^(1)^ containing the first mutation in *π*. During each iteration, the best tree *t*^(*ℓ*)^ is popped from the queue and extended. The extensions are scored and reintroduced into the queue. Only the *k* trees with the highest scores in the queue are kept, while others are discarded. The bars next to each genotype matrix indicate its perturbed log probability, *G*_*ϕ*_. Bars with grey fill correspond to the top-*k* trees that are retained and extended. Genotype matrices within dashed boxes denote parts of the search space that are not explored further. Orchard’s best reconstructed tree can then be input into the phylogeny-aware clustering algorithm. This algorithm conducts agglomerative clustering on the mutation trees to produce a set of clone trees. Each clone tree’s set of clones is scored, and the algorithm yields the clone tree that minimizes the Generalized Information Criterion (GIC). See Section 2.6 and Section A4 in S1 Appendix for complete details.

Orchard performs beam search in a search tree to generate *k* samples without replacement from *Q*^*π*^(*B*|*D*). In this search tree, each node represents a unique mutation tree, leaf nodes are complete mutation trees, and internal nodes are partial mutation trees (see Fig 2). Each node in the search tree is labeled with the perturbed (unnormalized) log probability of its corresponding mutation tree. The search tree has a total of (*n* + 1)^*n*−1^ leaves, representing each unique mutation tree that can be constructed from *n* mutations. Orchard’s search routine finds the top-*k* leaves of the search tree with the largest perturbed log probabilities without exhaustive exploration [38]. To further reduce run times, Orchard employs heuristic optimizations that, empirically, do not compromise its accuracy.

#### 2.5.1 Background on Ancestral Gumbel-Top-k trick

Sampling by finding the largest perturbed log probabilities is an example of a *Gumbel-Max trick*. These tricks derive from the motivating observation that if *ϕ*_*i*_ is the unnormalized log probability of the *i*-th item in a set, {*ϕ*_1_, …, *ϕ*_*f*_}, and *G*^(*i*)^ ∼ Gumbel(0, 1) is an i.i.d. sample from the standard Gumbel distribution—where Gumbel(*μ*, *β*) has a location parameter *μ* and a scale parameter *β*—then the following relationship holds:

$$\begin{array}{c} I=\underset{i}{\text{arg}\;\text{max}}\{\phi_{i}+G^{\left(i\right)}\}∼\text{Categorical}(\frac{\text{exp}(\phi_{i})}{\sum_{j=1}^{f}\text{exp}\left(\phi_{j}\right)},i\in \left\{1,2,\ldots ,f\right\}). \end{array}$$ (10)

Eq 10 is recognized as the original Gumbel-Max trick [42], and we refer to the quantities $G_{\phi_{i}}=\phi_{i}+G^{\left(i\right)}$ as *perturbed log probabilities*, noting that $G_{\phi_{i}}∼\text{Gumbel}\left(\phi_{i},1\right)$. It shows that finding the index of the largest perturbed log probability in a set is equivalent to sampling from a categorical distribution, defined by unnormalized log probabilities associated with each element. This powerful observation can be used to convert various combinatorial optimization algorithms into sampling algorithms. In a simple example, one can draw *k* samples without replacement from {*ϕ*_1_, …, *ϕ*_*f*_}, using an extension of the Gumbel-Max trick called the *Gumbel-Top-k* trick [39], by finding the *k* largest perturbed log probabilities in the set $\left\{G_{\phi_{1}},G_{\phi_{2}},\ldots ,G_{\phi_{f}}\right\}$ by, e.g., sorting the set [39].

Applying Gumbel-Top-k to sample from either *P*(*B*|*D*) or *Q*^*π*^(*B*|*D*) would require instantiating all (*n* + 1)^*n*−1^ unique trees over *n* mutations. Fortunately, the conditional independence structure of *Q*^*π*^(*B*|*D*) supports a tractable extension called the *Ancestral Gumbel-Top-k* trick. The Ancestral Gumbel-Top-k trick can be used alongside a branch-and-bound search to find the highest-scoring trees, leveraging a property of Gumbel variables known as *max-stability*. Specifically, the maximum of a set of independent Gumbel random variables $\left\{G_{\phi_{1}},G_{\phi_{2}},\ldots ,G_{\phi_{f}}\right\}$ follows a Gumbel distribution with a location equal to the log-sum-exp of their log probabilities {*ϕ*_1_, *ϕ*_2_, …, *ϕ*_*f*_} [38, 42, 43], i.e.,

$$\begin{array}{c} \underset{i\in \{1,2,\ldots ,f\}}{\text{max}}\;G_{\phi_{i}}∼\text{Gumbel}(\text{log}\sum_{i=1}^{f}\;\text{exp}\left(\phi_{i}\right),1). \end{array}$$ (11)

Max-stability can be used to determine the maximum perturbed log probability in a subtree of the search tree. This property allows a branch-and-bound beam search to efficiently find the top *k* largest perturbed log probabilities in the search tree, avoiding exhaustive exploration. To illustrate this, first, let *B*^(*ℓ*)^ be a genotype matrix with log probability *ϕ* = log *Q*^*π*^(*B*^(*ℓ*)^|*D*^(*ℓ*)^), and let *B*^(*ℓ*+1,*i*)^ be its *i*-th extension (out of *f* possible ones) with log probability *ϕ*_*i*_ = log *Q*^*π*^(*B*^(*ℓ*+1,*i*)^|*D*^(*ℓ*+1)^). Note that *ϕ* is the log-sum-exp of the set ${\left\{\phi_{i}\right\}}_{i=1}^{f}$:

$$\begin{array}{c} \text{exp}\left(\phi \right)=Q^{\pi }\left(B^{\left(\ell \right)}|D^{\left(\ell \right)}\right)=Q^{\pi }\left(B^{\left(\ell \right)}|D^{\left(\ell +1\right)}\right) \end{array}$$ (12)

$$\begin{array}{c} =\sum_{i=1}^{f}P\left(B^{\left(\ell +1,i\right)}|B^{\left(\ell \right)},D^{\left(\ell +1\right)}\right)Q^{\pi }\left(B^{\left(\ell \right)}|D^{\left(\ell \right)}\right) \end{array}$$ (13)

$$\begin{array}{c} =\sum_{i=1}^{f}\text{exp}\left(\phi_{i}\right), \end{array}$$ (14)

where Eqs 13 and 14 result from marginalizing over all *f* extensions of *B*^(*ℓ*)^. Thus we can write

$$\begin{array}{c} \underset{i\in \{1,2,\ldots ,f\}}{\text{max}}\;G_{\phi_{i}}=G_{\phi }∼\text{Gumbel}\left(\phi \right). \end{array}$$ (15)

So, if computing *ϕ* is tractable, then one can determine the maximum perturbed log probability of the set ${\left\{\phi_{i}\right\}}_{i=1}^{f}$ without computing any of the *ϕ*_*i*_s. Additionally, because Eq 15 can be applied recursively to each extension of *B*^(*ℓ*)^, and so on, down to the leaves of the search tree rooted at *B*^(*ℓ*)^, the perturbed log probability of *B*^(*ℓ*)^ is also the maximum of any complete mutation trees that have *B*^(*ℓ*)^ as a submatrix.

Orchard leverages this property by using *G*_*ϕ*_ as an estimate of the best complete tree that can be found by sampling the leaves in the subtree below *B*^(*ℓ*)^ and employs a beam search to explore only those parts of the search tree containing the top-*k* leaves.

#### 2.5.2 Computing the “shifted” perturbed log probabilities of extensions

When Orchard extends *t*^(*ℓ*)^, it has already sampled *G*_*ϕ*_. For each extensions *t*^(*ℓ*+1,*i*)^ that’s considered, $G_{\phi_{i}}$ is sampled. However, each $G_{\phi_{i}}$ must be sampled conditionally such that $G_{\phi }={\text{max}}_{i\in \{1,2,\ldots ,f\}}G_{\phi_{i}}$ (Eq 15). Orchard achieves this by initially sampling the set $\left\{G_{\phi_{1}},G_{\phi_{2}},\ldots ,G_{\phi_{f}}\right\}$, then applying a transformation, as described in [38], to ensure this condition is satisfied, resulting in a new set $\left\{{\bar{G}}_{\phi_{1}},{\bar{G}}_{\phi_{2}},\ldots ,{\bar{G}}_{\phi_{f}}\right\}$. We briefly describe this transformation here, but encourage the reader to refer to [38] for a complete proof of correctness. Let $Z={\text{max}}_{j}\;G_{\phi_{j}}$ and $T={\bar{G}}_{\phi }$. We assume ${\bar{G}}_{\phi }$ is also readily available, as it can be initialized to 0 at the start of the algorithm. The transformation sets *Z* = *T*, and shifts each $\left\{G_{\phi_{1}},G_{\phi_{2}},\ldots ,G_{\phi_{f}}\right\}$ to be less than or equal to *Z* using the truncated Gumbel CDF:

$$\begin{array}{cc} {\bar{G}}_{\phi_{i}} & =F_{\phi_{i},T}^{-1}\left(F_{\phi_{i},Z}\left(G_{\phi_{i}}\right)\right)\\ & =\text{log}\left(\text{exp}\left(-T\right)\right)-\text{exp}\left(-Z\right)+\text{exp}\left(-G_{\phi_{i}}\right), \end{array}$$ (16)

where ${\bar{G}}_{\phi_{i}}$ is the “shifted” perturbed log probability of the *i*-th extension, *F*_*ϕ*,*T*_(*g*) is the CDF of the Gumbel distribution truncated at *T*, and $F_{\phi ,T}^{-1}\left(u\right)$ is the inverse CDF of the Gumbel distribution truncated at *T*. Please see [38] for a numerically stable version of Eq 16.

#### 2.5.3 Stochastic beam search

Algorithm 1 contains the pseudocode for the Orchard algorithm. Orchard’s search routine can be considered a *stochastic beam search*, and is adapted from the algorithms described in [37, 38]. Orchard keeps track of partial trees using a queue with a fixed size *k*, a user-defined parameter called the *beam width*. At each iteration, the partial tree *t*^(*ℓ*)^ with the largest ${\bar{G}}_{\phi }$ is popped from the queue and extended. For each extension *t*^(*ℓ*+1,*i*)^ considered, we compute a perturbed log probability, but to do so, we need to compute, or estimate, *P*(*D*^(*ℓ*+1)^|*B*^(*ℓ*+1,*i*)^), which is a computationally intensive operation. As such, we use a heuristic, denoted by the function *H*(*t*^(*ℓ*)^, *f*), which uses an analytical approximation to score all extensions based on their adherence to the ISA, and selects the top-*f* extensions based on these scores. The parameter *f* is user-defined, and is called the *branching factor*. In practice, we find this heuristic decreases Orchard’s run time without compromising its accuracy. Complete details for this heuristic are provided in Section A3.1 in S1 Appendix.

**Algorithm 1**: Orchard

 **input**: A dataset *D* containing allele frequency data and copy number states for

    *n* mutations in *m* tissue samples

 **parameters**: k (*beam width*), f (*branching factor*), *π* (*mutation order*)

 **output**: $t_{1}^{*},t_{2}^{*},\ldots ,t_{k}^{*}$, the top-*k* trees

 // Initialize first tree assuming that $\pi =[\begin{array}{cccc} 1 & 2 & \ldots & n \end{array}]$

1 $t^{\left(1\right)}=\{V^{\left(1\right)}=\left\{r,1\right\},E^{\left(1\right)}=\{(r,1)\},M^{\left(1\right)}=[\begin{array}{cccc} 1 & -1 & \ldots & -1 \end{array}]\}$

2 ${\bar{G}}_{\phi }=0,G_{\phi }=0$ // initialize scores

3 $Q=[(t^{\left(1\right)},{\bar{G}}_{\phi },G_{\phi })]$

4 **while** *Q is not empty* **do**

5  $(t^{\left(\ell \right)},{\bar{G}}_{\phi },G_{\phi })=\;\text{take}\;\text{and}\;\text{remove}\;\text{the}\;\text{first}\;\text{element}\;\text{of}\;Q$

   // H(*t*^(*ℓ*)^, *f*) returns a set of *f* indices corresponding to extensions of *t*^(*ℓ*)^ with the best adherence to the ISA—this helps speed up lines 14, 16

6  $S=\{G_{\phi_{i}}=\phi_{i}+G^{\left(i\right)}:i\in \text{H}\left(t^{\left(\ell \right)},f\right)\}$

7  **if** *ℓ* + 1 == *n* **then**

8   *k* = *k* − 1

9   **yield** t* = arg max_*i*_ *S* // yield best tree in *S*

10   Return to line 5

11  **else**

   // “Shift” perturbed log probabilities using Eq 16

12   *Z* = max_*i*_ *S*

13   *T* = *G*_*ϕ*_

14   $\bar{S}=\{{\bar{G}}_{\phi_{i}}=\text{log}(\text{exp}\left(-T\right)-\text{exp}\left(-Z\right)+\text{exp}\left(-G_{\phi_{i}}\right)):G_{\phi_{i}}\in S\}$

15   $\text{add}\;\text{each}\;(t^{\left(\ell +1\right)},{\bar{G}}_{\phi_{i}},G_{\phi_{i}})\;\text{to}\;Q$

16   Sort *Q* in descending order according to ${\bar{G}}_{\phi }$, discard all but top-*k*

#### 2.5.4 Approximating the MLE of the clonal proportion matrix

Ideally, if we seek to score a mutation tree represented by its binary genotype matrix *B*, we would integrate out the clonal proportions *U*. In this case, the data likelihood for *B*, *P*(*D*|*B*), assuming a uniform prior on *U*, is given by

$$\begin{array}{c} P\left(D|B\right)\propto \prod_{s}\int_{0}^{1\geq \sum_{v}U_{vs}}P\left(D_{s}|B,U_{:s}\right)dU_{:s}, \end{array}$$ (17)

where *D*_*s*_ are the data associated with sample *s* and the integration is over non-negative values of *U*_*vs*_ such that ∑_*v*_*U*_*vs*_ ≤ 1. We know of no analytical solution to this integral, instead we use a point estimate of *U*, U* and use the approximation

$$P\left(D|B\right)\approx \prod_{s}P\left(D_{s}|B,U_{:s}^{*}\right).$$

A good point estimate of *U* would be its maximum likelihood estimate (MLE) given *D* and *B*. Instead, to speed up Orchard, we use the projection algorithm [44, 45] which finds a point estimate U* by optimizing the following quadratic approximation to the binomial log likelihood of *U* given *B* and *D*:

$$\begin{array}{c} LP\left(U^{*};B,\hat{F},W\right)=\underset{F^{*},U^{*}}{\text{min}}\;\|W⊙(\hat{F}-F^{*}){\|}^{2}\;\text{s.t.}\;1^{T}U^{*}\leq 1,U^{*}\geq 0,F^{*}=BU^{*}, \end{array}$$ (18)

where ‖⋅‖ is the Frobenius norm, **1** is a vector of “1”s, $\hat{F}$ are the observed mutation frequencies, *W* is an *n* × *m* matrix of inverse-variances for each mutation in each sample derived from *D*, and ⊙ is the Hadamard, i.e., element-wise product. Further details on computing the elements of *W* and U* can be found in Section A2.3 in S1 Appendix.

#### 2.5.5 Approximating the complete data likelihood

Orchard evaluates a genotype matrix *B* and its corresponding clonal proportion matrix *U* using an approximation of the complete data likelihood of the input data *D*. This approximation assumes that the observed bulk read count data for each locus is generated according to a binomial sampling model, i.e.,

$$\begin{array}{c} b_{js}∼\text{Binom}\left(N_{js},\lambda_{js}\right), \end{array}$$ (19)

where *b*_*js*_ is the count of reads containing the variant allele *j* in sample *s*, *N*_*js*_ = *a*_*js*_ + *b*_*js*_ is the total number of reads mapped to the locus containing the variant allele *j* in sample *s*, and λ_*js*_ is the variant allele frequency for the variant allele *j* in sample *s*. We do not observe λ_*js*_, but instead infer it from the mutation frequency matrix *F*. Given a genotoype matrix *B* and a clonal proportion matrix *U*, we calculate the mutation frequency matrix with *F* = *BU* (Eq 1). We can use the following formula to estimate the variant allele frequency that generated the observed bulk read count data for mutation *j* in sample *s*:

$$\begin{array}{c} \lambda_{js}=F_{js}\omega_{js}, \end{array}$$ (20)

where λ_*js*_ is the inferred variant allele frequency, *F*_*js*_ is the frequency of mutation *j* in sample *s*, and *ω*_*js*_ is the variant read probability provided as an input along with the read count data. We then compute the approximate complete data likelihood as follows:

$$\begin{array}{c} P\left(D|B,U\right)\approx \prod_{s}L(s,F_{:s}) \end{array}$$ (21)

$$\begin{array}{c} L\left(s,F_{:s}\right)=\prod_{v}\text{Binom}(b_{vs}|a_{vs}+b_{vs},\omega_{vs}F_{vs}). \end{array}$$ (22)

We assume that the samples are exchangeable in Eq 21. To complete our definition for Eq 22, we define Binom(b|N,λ)=(Nb)λb(1-λ)a as the binomial probability of observing *b* variant reads out of a total of *N* = *a* + *b* reads mapping to its locus when the variant allele frequency is λ.

### 2.6 Phylogeny-aware clustering

Orchard contains a “phylogeny-aware” clustering algorithm that uses a reconstructed mutation tree and its corresponding mutation frequency matrix *F* to cluster mutations into subclones and resolve clone trees. This algorithm performs agglomerative clustering and joins adjacent nodes *u* and *v* in a tree using the following formula:

$$\begin{array}{cc} {\text{min}}_{u,v}d(u,v),\\ d(u,v) & =\frac{n_{u}n_{v}}{n_{u}+n_{v}}{\left\|{\bar{F}}_{u}-{\bar{F}}_{v}\right\|}^{2}, \end{array}$$

where *n*_*u*_ is the number of mutations associated with node *u*, ${\bar{F}}_{u}$ is the average mutation frequency of the mutations contained in node *u*, and ‖⋅‖ is the Euclidean norm. This definition for *d*(*u*, *v*) is known as *Ward’s method*. The agglomerative clustering is repeated, successively joining adjacent nodes, until it reaches a clone tree consisting of only the root node and a single node containing all mutations. Note that only parent-child pairs, where the child is the parent’s only child, are considered for joining. Once no such pairs remain, any adjacent nodes can then be joined. The algorithm outputs *n* different clusterings containing 1, …, *n* clones. Each clone tree containing *c* clones has a corresponding *n* × *c* binary matrix *Z*, where *Z*_*ji*_ = 1 if and only if mutation *j* originated in clone *i*. Associated with each clone *i* in each sample *s* is its variant allele frequency, λ_*is*_. If mutation *j* is assigned to clone *i*, then it is assumed that the observed variant allele frequency for mutation *j* in sample *s*, ${\hat{\lambda }}_{js}$, is a noisy observation of λ_*is*_, such that ${\hat{\lambda }}_{js}=\lambda_{is}+\epsilon$, where *ϵ* is a noise term. So, given *F*, *Z*, and *D*, the likelihood of a clustering can be expressed as:

$$\begin{array}{c} L\left(F|D,Z\right)=\sum_{i=1}^{c}\sum_{j=1}^{n}I\left(Z_{ji}=1\right)\sum_{s=1}^{m}\;\text{log}\;(\text{Binom}(b_{js}|N_{js},\lambda_{is})), \end{array}$$ (23)

where Binom(b|N,λ)=(Nb)λb(1-λ)a is defined as the binomial probability of observing *b* variant reads out of a total of *N* = *a* + *b* reads mapping to its locus when the variant allele frequency is λ, and $I(Z_{ji}=1)$ is the indicator function which evaluates to 1 if mutation *j* is assigned to clone *i*, and 0 otherwise. Maximizing Eq 23 may result in overfitting, where each mutation is assigned to its own clone. To mitigate this issue, the clone tree with the clustering that minimizes the Generalized Information Criterion (GIC) [46] is yielded. The implementation details of this algorithm are provided in Section A4 in S1 Appendix.

## 3 Results

### 3.1 Evaluation overview

We compare Orchard with two state-of-the-art MSPP reconstruction algorithms: CALDER [26], and Pairtree [25]. For CALDER we used its non-longitudinal model and v8.0.1 of the Gurobi optimizer. CALDER only outputs a single tree which is optimal under its mixed integer linear programming formulation, or otherwise fails to output a valid tree. Pairtree uses Markov Chain Monte Carlo to sample trees from a data-implied posterior over trees. We set Pairtree’s *trees-per-chain* parameter to 5000, and otherwise used the default parameters. We ran the Pairtree algorithm in parallel on 32 CPU cores, generating 160000 trees (32 × 5000). All Orchard experiments used the same branching factor *f* = 20, but we evaluated two beam widths: *k* = 1, and *k* = 10. We also ran the Orchard algorithm in parallel on 32 CPU cores; generating 32 trees (32 × 1) and 320 trees (32 × 10), respectively. Parallel instances of Orchard sample trees independently, so we are only guaranteed a minimum of 1 or 10 unique trees, respectively. Running parallel instances of Orchard and Pairtree means that each algorithm was effectively executed 32 times on each dataset, with each parallel instance having a unique random seed. CALDER, being deterministic when run on identical computing hardware with the same inputs, requires only a single run to obtain its best reconstruction.

We assess the mutation frequency matrix *F* and genotype matrix *B* for the tree(s) output by a MSSP reconstruction method by comparing them with a baseline mutation frequency matrix *F*^(baseline)^ and, when available, a set of ground-truth genotype matrices. For simulated bulk data, *F*^(baseline)^ corresponds to the ground-truth mutation frequency matrix *F*^(true)^ used to generate the simulated VAF data. For real bulk data where an expert-defined clone tree is available, *F*^(baseline)^ corresponds to a *maximum a posteriori* (MAP) mutation frequency matrix *F*^(MAP)^ that we fit to the expert-defined tree (see Section A5.2 in S1 Appendix). Each mutation in *F*^(MAP)^ is assigned a frequency according to the MAP mutation frequency of the subclone the mutation belongs to in the expert-defined clone tree. We compare the reconstructed mutation frequencies in *F* to *F*^(baseline)^ using a metric called the *log perplexity ratio* [25]. The log perplexity ratio is the log of the ratio between the perplexity of *F* and the perplexity of *F*^(baseline)^. A negative log perplexity ratio indicates that the mutation frequency matrix *F* for the tree(s) reconstructed by a method agree with the VAF data better than *F*^(baseline)^. Perplexity is evaluated under a binomial probability model. We compare the reconstructed genotype matrix *B* to the set of ground-truth genotype matrices using a metric called the *relationship reconstruction loss* [25]. The relationship reconstruction loss compares the evolutionary relationships in *B* to those in the set of ground-truth genotype matrices using the Jensen-Shannon divergence. The log perplexity ratio and relationship reconstruction loss are computed as likelihood-weighted averages over all distinct trees returned by a method. This approach improves the robustness and reliability of our evaluations by incorporating the uncertainty in the reconstructions. We also evaluate MSSP reconstruction methods based on their *wall clock runtime*, representing the time (in seconds) each method requires to complete on a data set. For more details on these metrics see Section A5 in S1 Appendix.

### 3.2 Orchard produces better reconstructions than competing methods on 90 simulated cancers

We used the Pearsim software (https://github.com/morrislab/pearsim) from [25] to generate 90 simulated mutation trees and corresponding VAF data which adheres to the ISA. The data sets vary in the number of mutations (10, 30, 50, 100, 150, 200), cancer samples (10, 20, 30, 50, 100), and sequencing depths (50x, 200x, 1000x). These simulations all adhere to the ISA.

**Fig 3:**
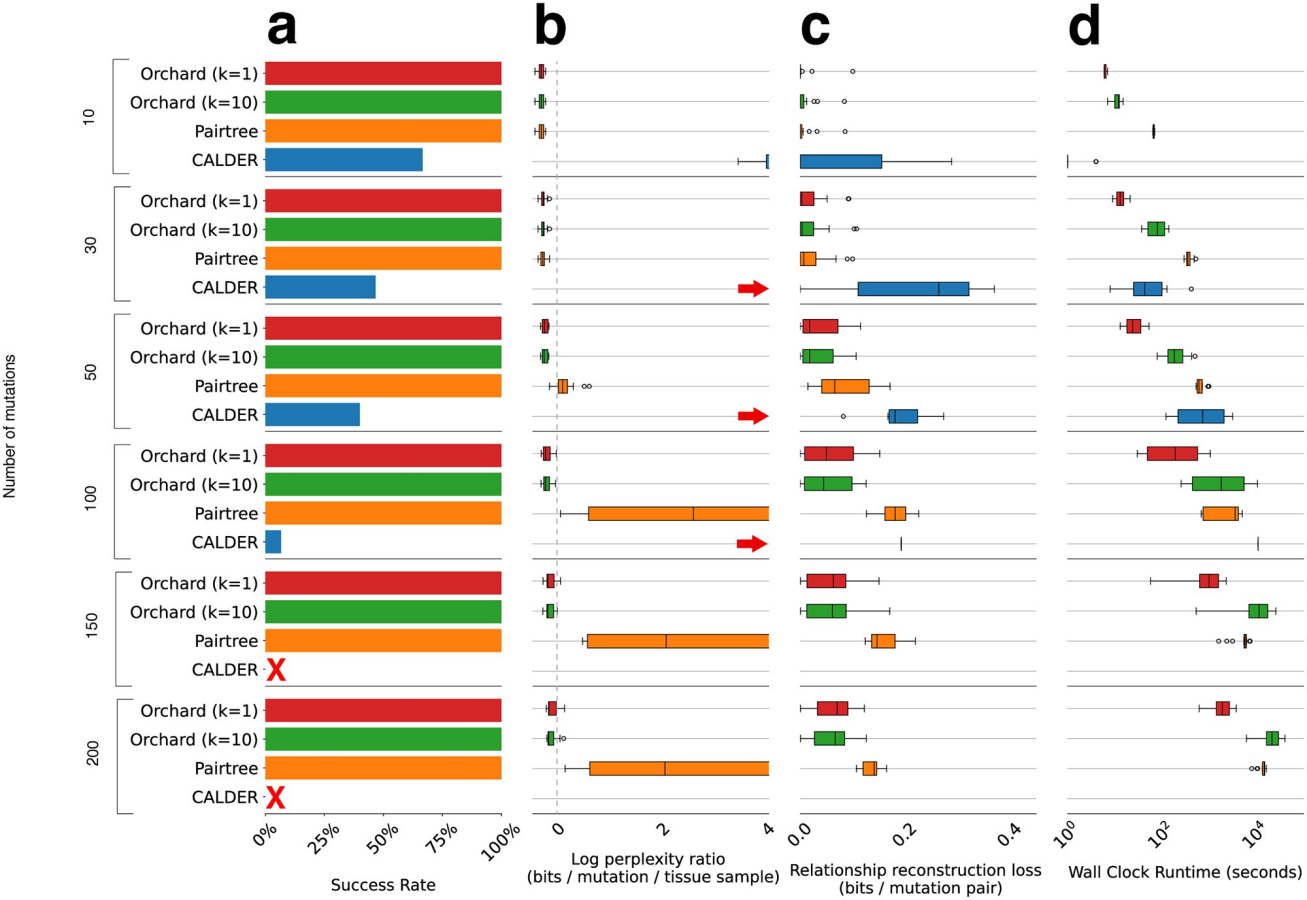
Evaluation of reconstructions for 90 simulated mutation trees. Results are grouped by the size of the simulated mutation trees (rows), i.e., the *problem size*. **a**. Bar plots show the percentage of data sets where a method produces at least one valid tree. A red *x* means the method did not succeed on any of the data sets for that problem size. A red arrow means the results for the method on a problem size occur beyond the x-axis limit. The distributions, represented by box plots, in (b,c,d) only include data sets where the method was successful. **b**. The distribution of log perplexity ratios, a measure of VAF data fit. Ratios are relative to the perplexity of the ground truth mutation frequency matrix *F*^(true)^, and can be negative. Lower log perplexity ratios indicate better reconstructions. **c**. The distributions of relationship reconstruction loss for each method on a problem size. This loss can range between zero bits (complete match of pairwise relationships) and one bit (complete mismatch of pairwise relationships). **d**. The distributions of wall clock run time.

We ran Orchard, CALDER, and Pairtree on each simulated data set a single time, and the box plots in Fig 3b, 3c and 3d show the distribution of results for each evaluation metric. Table 1 contains counts of data sets, separated by problem size, where a method had the best log perplexity ratio (P) or relationship reconstruction loss (R). CALDER was the only method to fail on a reconstruction problem, its failure rate increased as the problem size increased, and it failed to produce a valid tree on any reconstruction problem with more than 100 mutations.

**Table 1:** Count of simulated mutation tree data sets, out of 15 per column, where a model had the best log perplexity ratio (P) or relationship reconstruction loss (R). Bold indicates column max.

	Simulated data set size (mutations)											
	10		30		50		100		150		200	
	*P*	*R*	*P*	*R*	*P*	*R*	*P*	*R*	*P*	*R*	*P*	*R*
CALDER	0	1	0	0	0	0	0	0	0	0	0	0
Pairtree	1	0	4	3	0	0	0	0	0	0	0	0
Orchard (k = 1)	**8**	**13**	**8**	**9**	**10**	5	4	7	1	**8**	4	7
Orchard (k = 10)	6	1	3	3	5	**10**	**11**	**8**	**14**	7	**11**	**8**

Fig 3 and Table 1 illustrate a problem-size-dependency in relative performance. Orchard generally outperformed Pairtree and CALDER on the small reconstruction problems (10–30 mutations) on all metrics. Although Orchard’s reconstructions are only slightly better than Pairtree for these problems, Orchard is 5–10x faster. In contrast, Orchard consistently outperformed Pairtree and CALDER on all problems with 50 or more mutations. For these problems, in either setting of *k*, Orchard finds trees that are significantly better than those found by Pairtree or CALDER. Unfortunately, the run time required by Orchard increases significantly on larger problem sizes. Detailed run time breakdowns for each method across problem sizes can be found in Table D in S1 Appendix. Orchard (*k* = 1) generally outperforms Orchard (*k* = 10) on smaller problems (10–30 mutations), but this switches on larger problems. To understand this change, we inspected the trees recovered by Orchard (*k* = 1 and *k* = 10). In nearly all 10 or 30-mutation tree reconstruction problems, both Orchard configurations found the same best tree. However, Orchard (*k* = 10) yields nine other sub-optimal trees which are included in the weighted averages used to compute the metrics resulting in slightly worse average scores. On larger problems, the trees reconstructed by Orchard (*k* = 10) are generally much better than those reconstructed by Orchard (*k* = 1).

### 3.3 Orchard reconstructs more plausible trees on 14 B-progenitor acute lymphoblastic leukemias

We applied Orchard, CALDER and Pairtree on real bulk data from 14 B-progenitor acute lymphoblastic leukemias (B-ALLs) originally studied in [47]. The B-ALL data sets vary in the number of subclones (between 5 and 17), represented by expert-defined subclones, tissue samples (between 13 and 90), and mutations (between 16 and 292). All of the B-ALL data sets had an approximate sequencing depth of 212x [47]. Each B-ALL patient had samples taken at diagnosis and relapse, and then each of these samples was transplanted into immunodeficient mice resulting in multiple patient derived xenografts (PDXs). The VAF data for each B-ALL was used by experts to manually cluster mutations into subclones and construct clone trees, both of which were reviewed for biological plausibility [47].

Table A in S1 Appendix presents a comprehensive comparison of each method’s log perplexity ratios on the 14 B-ALL clone tree reconstruction problems. Orchard generally outperformed Pairtree, achieving a slightly lower log perplexity ratio on 10/14 data sets, with an average reduction of -2.17e-5 bits. CALDER, on the other hand, failed on 2 of the data sets and performed the worst on those it did succeed on. It is unsurprising that both Orchard and Pairtree perform well on the B-ALL data, given that each data set contains at least 13 samples, and at most 17 clones. Typically, as the number of samples increases, the number of plausible trees typically decreases, making accurate reconstruction more feasible [25].

In most practical scenarios, expert-defined subclones are unavailable. To emulate these conditions using the B-ALL data, we evaluate two approaches:

1. The traditional approach of clustering mutations into subclones using VAF-based clustering algorithms and then constructing a clone tree from these subclones.
2. Our new approach of reconstructing a mutation tree using the VAF data and subsequently utilizing this mutation tree to guide the inference of subclones.

We provide a detailed assessment of both approaches in Section A6.8 in S1 Appendix. To assess the second approach, we first reconstructed mutation trees for each of the 14 B-ALL data sets using both Orchard (k = 10) and Pairtree. We provide a comprehensive analysis of the mutation trees reconstructed by each method in Section A6.5 in S1 Appendix. Orchard and Pairtree reconstruct similarly accurate mutation trees on B-ALL data sets containing fewer than 50 mutations, but Orchard reconstructs significantly more plausible trees than Pairtree for B-ALLs with more than 50 mutations.

**Fig 4:**
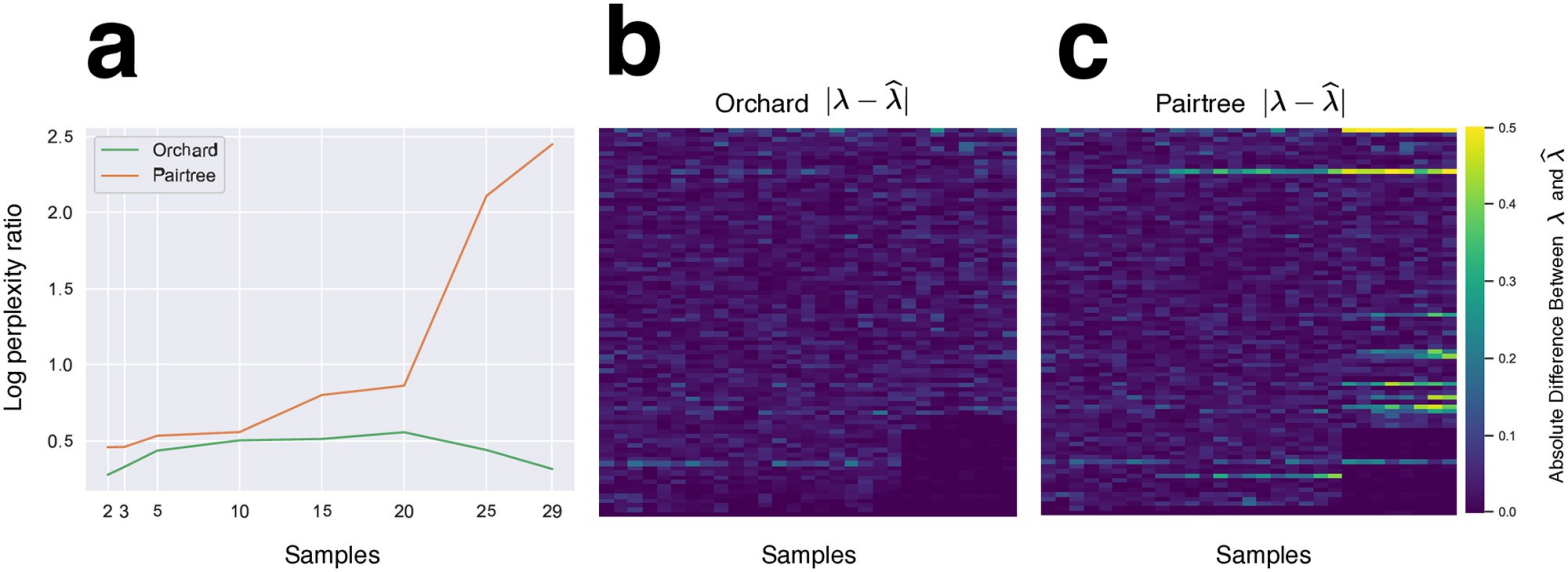
Evaluation of reconstructions by Orchard and Pairtree for SJBALL022611. **a**. Log perplexity ratio for the trees reconstructed by Orchard and Pairtree as a function of the number of samples. Orchard’s reconstructions are accurate regardless of the number of samples provided, while Pairtree’s reconstructions worsen with more samples. **b,c**. Absolute difference between the VAFs inferred by Orchard and Pairtree and the VAFs implied by the bulk data. Large values indicate divergence between VAFs inferred by a method and the VAFs implied by the data. VAFs inferred by Orchard adhere very closely to the data, while also adhering to the ISA. Pairtree’s poorly reconstructed tree results in innaccurate VAF estimates for many mutations. The same row in each heatmap corresponds to same unique mutation, and each column corresponds to the same unique sample.

The accuracy of Orchard’s mutation tree reconstructions, compared to Pairtree, is highlighted by further analyzing the B-ALL data set SJBALL022611. This data set has 84 mutations identified across 29 different samples. First, Orchard (k = 10) and Pairtree were used to reconstruct trees for this data using a varied number of samples (2,3,5,10,15,20,25,29). For each number of samples, we selected those with the largest number of variant reads. Fig 4a shows the log perplexity ratio for the trees reconstructed by Pairtree and Orchard when varying the number of samples used. The baseline mutation frequencies were chosen to be the MAP mutation frequencies, *F*^(MAP)^, fit to the expert-defined clone tree for SJBALL022611. Counter intuitively, Fig 4a demonstrates that as more samples are used during reconstruction, the mutation frequencies reconstructed by Pairtree exhibit poorer fit to the VAF data. This phenomenon was observed across all B-ALL data sets with more than 60 mutations, indicating that Pairtree’s reconstructions become increasingly less reliable with more data. In contrast, Orchard’s reconstructions exhibit excellent data fit irrespective of the number of mutations or samples. In Section A6.6 in S1 Appendix, we conducted a similar experiment using random subsets of samples for reconstruction. The results of this experiment strongly align with those shown in Fig 4a.

The poor data fit for Pairtree’s best reconstructed tree for SJBALL022611 using all 29 samples is exemplified in Fig 4c. This figure presents a heatmap of the absolute differences between the VAFs inferred by Pairtree and those implied by the bulk data for each mutation, and it shows that Pairtree’s inferred VAFs significantly diverge from those implied by the bulk data, indicating a poor reconstruction. Again, in contrast, Fig 4b demonstrates that Orchard’s reconstructions fit the VAF data very well, as evidenced by the minimal difference between the data-implied VAFs and those inferred by Orchard. Comparing these two heatmaps reveals that Pairtree infers significantly larger differences than Orchard (confirmed by one-tailed t test *P* < 0.001).

Next, we use the phylogeny-aware clustering algorithm to infer clones from each method’s best tree that was reconstructed using all of the samples for SJBALL022611. We can compare the inferred clones to the expert-defined clones using the Adjusted Rand Index (ARI). The subclones inferred from Orchard’s tree exhibited an ARI of 0.96, indicating a near-perfect match with the expert-defined subclones. In contrast, the subclones inferred from Pairtree’s tree were more crude, obtaining a notably lower ARI of 0.82. With the exception of two B-ALL data sets (SJBALL022609, SJBALL022610), the subclones identified by the phylogeny-aware clustering algorithm using Orchard’s tree matched the expert-defined subclones as well or better than those identified using Pairtree’s tree. These results are consistent with our expectations, as we believe better tree reconstructions should be more informative for guiding clone inference. We provide a more detailed discussion of the phylogeny-aware clustering results for the B-ALL data in Section A6.7 in S1 Appendix.

## 4 Discussion

Modern cancer evolution studies generate large amounts of bulk sequencing data often containing hundreds of mutations and multiple cancer samples. This vast amount of data presents a promising opportunity to improve our understanding of the evolutionary events that drive cancer progression. Until now, existing mixed sample perfect phylogeny (MSPP) reconstruction algorithms were unable to reliably reconstruct trees with more than 30 mutations. These limitations necessitated clustering mutations into subclones prior to reconstructing trees, which is an error prone process [30–32]. We introduced Orchard, a novel MSPP reconstruction algorithm that uses variant allele frequency data to build large and accurate mutation trees. We derived an approximation to the mutation tree posterior, then showed how Orchard adapts Ancestral Gumbel-Top-k sampling to efficiently sample without replacements from it. Our results demonstrate that Orchard can reliably match or beat ground-truth (or expert-derived) baselines on tree reconstruction problems with up to 300 mutations and up to 100 samples from the same cancer. Orchard can recover larger trees on simulated data, and in Section A6.4 in S1 Appendix we use Orchard to successfully reconstruct 1000-node simulated mutation trees in ≈26 hours of wall clock time. Orchard’s ability to reconstruct extremely large trees motivated a new strategy where the mutation tree structure is used to guide the inference of clones and clone trees. To this end, we introduced a “phylogeny-aware” clustering algorithm that uses agglomerative clustering to infer clones and clone trees from mutation trees.

There are a few potential areas of improvement for Orchard. First, the majority of Orchard’s run time is used by the projection algorithm [44] to estimate the clonal proportion matrix *U* each time it adds a new mutation to a tree. Although the computational complexity for estimating *U* is, at worst, quadratic in the number of mutations (i.e., rows) in *U*, the wall clock times grows quickly with increasing problem size, beam width (*k*), or branching factor (*f*). One potential improvement, because the frequency of most mutations should not be changed by introducing a new mutation into the tree, is developing a new version of the projection algorithm that only performs local updates to the *U* matrix. Also, Orchard marginalizes over all possible ways to place a new mutation into a partially built tree, which requires an exponential number of evaluations when the tree is a star tree, i.e., a tree where all mutations are children of the root. Unless specified otherwise, Orchard will try all 2^*ℓ*^ + *ℓ* possible ways to place a new mutation into a star tree with *ℓ* mutations. Fortunately, under the ISA, such trees are uncommon and linear branching becomes more likely as the number of children for a node increases. So, in practice, it is rare for nodes to have large numbers of children.

The current implementation of the “phylogeny-aware” clustering algorithm uses a non-probabilistic merging strategy to identify clones in a mutation tree. Despite this, the algorithm already matches, or in some cases, outperforms state-of-the-art probabilistic VAF-based clustering algorithms on real data (see Section A6.7 in S1 Appendix). These results suggest that the mutation tree structure is useful for guiding the inference of clones and clones trees. We anticipate that improvements to the current algorithm to make it probabilistic, so that its merging choices are made based on an appropriate likelihood function, will result in even greater performance.

We further anticipate the development of new algorithms that utilize large mutations trees reconstructed by Orchard. For example, these trees could be used to study patterns of somatic mutations, recover lineage-specific mutation signature activities, or identify patterns of metastatic spread [17]. The Orchard algorithm could also be adapted to reconstruct mutation trees from single-cell data, though this requires updating Orchard’s noise model and sampling routine to accommodate single cell data.

## Supporting information

S1 Appendix Supplementary information. (PDF)
