# Supplementary material for: Orchard: Building large cancer phylogenies using stochastic combinatorial search
Source: PLoS Comput Biol. 2024 Dec 30;20(12):e1012653. doi: 10.1371/journal.pcbi.1012653 (PMC11723595; doi:10.1371/journal.pcbi.1012653)
Supplement: S1 Appendix — (PDF) [file pcbi.1012653.s001.pdf]

Supplementary Text – Orchard: building large cancer phylogenies using stochastic combinatorial search

Ethan Kulman[1,2], Rui Kuang[2], Quaid Morris*[1],

1 Computational and Systems Biology Program, Sloan Kettering Institute, New York, New York, USA
2 Department of Computer Science and Engineering, University of Minnesota, Minneapolis, Minnesota, USA

A1 Calculating Orchard's Inputs

In this section, we provide details for calculating inputs required by Orchard. The actual input file formats for Orchard are the same as Pairtree [1], and these inputs have been described in detail in previous protocols [2].

A1.1 Computing the variant read probability

The variant read probability for mutation j in sample s, denoted as $\omega_{js} \in [0,1]$, is a correction that maps from the frequency of cells containing the mutant allele j in the sample s to the associated variant allele frequency of j. Orchard requires some basic information about the bulk sample s to compute ω_{js}. First, Orchard needs to know M_{js}, the number of copies of the mutant allele in cells in s that contain it. Orchard assumes that all j-containing cells in s have the same number of j alleles. Also, we need to know N_{js}, the average number of copies of the genomic locus containing j among the cells in s. Given N_{js} and M_{js}, then

$$\omega_{js} = \frac{M_{js}}{N_{js}}. \tag{24}$$

Often cancer cells have different copy numbers at specific loci compared to normal cells. In that case, we set

$$N_{js} = \rho_s N_{js}^{(c)} + (1 - \rho_s) N_{js}^{(h)},$$

where

- $N_{js}^{(c)}$ is the population average copy number of the locus containing the mutant allele j in the cancerous cell population,
- $N_{js}^{(h)}$ is the copy number of the locus containing the mutant allele j in the healthy cell population. For autosomes, $N_{js}^{(h)} = 2$, and
- ρ_s is the fraction of cells in bulk sample s that are cancerous, this is also known as the purity.

The values ρ_s and $N_{js}^{(c)}$ are outputs of CNA calling pipelines, and so should always be available. Computing M_{js} can be more challenging, we suggest, due to the ISA, using $M_{js} = 1$ unless there is strong evidence that the j-allele has been amplified. In that case, allele-specific CNA callers provide the multiplicity of the major allele A_{js} and the minor allele B_{js}, where $N_{js}^{(c)} = A_{js} + B_{js}$. In this case $M_{js} = A_{js}$ may be appropriate if A_{js} is a whole number. However, if a locus has many CNAs across the available samples, accurately estimating M_{js} can be challenging because of the possibly of subclonal

changes in j's multiplicity. In such scenarios, it may be necessary to exclude point mutations within genomic regions significantly affected by CNAs. For more details on estimating M_{js} and N_{js}, please see [3].

A1.2 Supervariant approximation

If Orchard is used to construct a clone tree, then it expects as input a set of mutation clusters representing individual clones. Orchard treats all mutations in the same clone as a single mutation or alternatively a "supervariant". A "supervariant" is a concept originally from [1] that approximates the data for a set of mutations C as a single mutation. We provide a brief explanation of this approximation scheme but refer the reader to [1] for complete details.

A "supervariant" approximation represents a group of mutations as a single mutation that can be added to a mutation tree. If C is a finite set of mutations, then the "supervariant" approximation of C combines the data of each mutation $j \in C$ in each samples $s = 1, \dots, m$, such that the data for C in sample s can be represented as $X_{Cs} = (a_{Cs}, b_{Cs}, \omega_{Cs})$, where a_{Cs}, b_{Cs}, and ω_{Cs} are the reference read count, variant read count, and variant read probability for C in sample s, respectively. One issue when combining the mutation data in C is that it is not guaranteed that all mutations $j \in C$ have the same variant read probability in sample s, ω_{js}. To resolve this, $\omega_{Cs} = \frac{1}{2}$ is fixed for all samples $s = 1, \dots, m$, and the data for all mutations $j \in C$ is scaled as follows:

$$
\begin{aligned}
T_{js} &= a_{js} + b_{js} \\
\tilde{T}_{js} &= 2\omega_{js}T_{js} \\
\tilde{b}_{js} &= min(b_{js}, \tilde{T}_{js}) \\
\tilde{a}_{js} &= \tilde{T}_{js} - \tilde{b}_{js} \\
\tilde{\omega}_{js} &= \frac{1}{2}
\end{aligned}
$$

After scaling the data for each mutation $j \in C$ for all samples $s = 1, \dots, m$, we can compute the values for X_{Cs} as follows:

$$
\begin{aligned}
X_{Cs} &= \{a_{Cs}, b_{Cs}, \omega_{Cs}\} \\
a_{Cs} &= round\left(\sum_j \tilde{a}_{js}\right) \\
b_{Cs} &= round\left(\sum_j \tilde{b}_{js}\right) \\
\omega_{Cs} &= \frac{1}{2}
\end{aligned}
$$

There are a few problems with the "supervariant" approximation. First, using the "supervariant" approximation results in a slightly different reconstruction problem than a mutation tree reconstruction problem, where the perfect phylogeny matrix B and the clonal proportion matrix U are inferred for the "supervariants" instead of the mutations. Also, grouping mutations with different variant read probabilities means that we assume mutations co-occur even though they are in genomic regions that vary in copy number. There is, in fact, uncertainty about whether or not these mutations are part of the same clone. Since Orchard can reconstruct extremely large trees, we could instead use a *relaxed "supervariant" approximation*, where pre-defined mutation clusters are split

apart such that only mutations with the same variant read probability appear in the same clone. This relaxation would remove some of the pitfalls introduced by the "supervariant" approximation. For simplicity, we chose to use the "supervariant" approximation as described here.

A2 Factorized approximation to the mutation tree posterior

In this section, we provide further details on the factorized approximation to the mutation tree posterior presented in Eq 5.

A2.1 Computing a mutation order

The factorized approximate posterior, $Q^{\pi}(B|D)$, relies on a mutation order π. We can design an order where mutations precede their descendants by ensuring the order adheres to the *sum constraint* [4,5] implied by the ISA. For a mutation order to adhere to the sum constraint it must be the case that:

$$u \succ v : \widehat{F}_{us} \geq \widehat{F}_{vs} \ \forall s,$$

i.e., mutation u must come before mutation v in the order if its frequency is at least as large in each sample. If the frequencies of u and v are the same across all samples, then either ordering $u \succ v$ or $v \succ u$ adheres to this constraint. If a mutation order can be found that agrees with the sum constraint, then each mutation could be added to the tree as a leaf node. However, $\widehat{F}$ often contains noise, and it's possible that no such order can be found. Instead, we relax the sum constraint and find a mutation order that adheres to the $\widehat{F}$-sum constraint:

$$u \succ v : \gamma_u \geq \gamma_v,$$

where $\gamma_w = \sum_{s=1}^{m} \widehat{F}_{ws}$. This order can always be found, regardless of whether or not $\widehat{F}$ contains noise, and if $\widehat{F}$ is noise free and the data adheres to the ISA, then this order will also adhere to the sum constraint. Orchard sets π according to the $\widehat{F}$-sum constraint, and since this ordering does not guarantee that mutations precede their descendants, Orchard considers all possible mutation placements, both as internal nodes and leaf nodes.

In some cases, the $\widehat{F}$–sum mutation order guarantees that mutations are placed in the tree before their descendants. When this holds true, $Q^{\pi}(B|D)$ can accurately approximate $P(B|D)$, as observing just $D^{(\ell+1)}$ may be sufficient for constructing an optimal partial tree. However, there are counter examples where, even if mutations are added to the tree before their descendants, the partial tree structure may preclude the discovery of the optimal tree. One example is when there are many equally plausible partial tree structures. To find the optimal complete tree, it may be necessary to observe the data for the last $n - \ell + 1$ mutations to determine which partial tree leads to it. This can require exhaustive exploration of the search space and an unbounded queue. Fig E shows an example of this.

A2.2 Alternative techniques for sampling from the approximate posterior

In this section, we discuss alternative techniques to sample from $Q^{\pi}(B|D)$. These techniques include a greedy approach and categorical sampling.

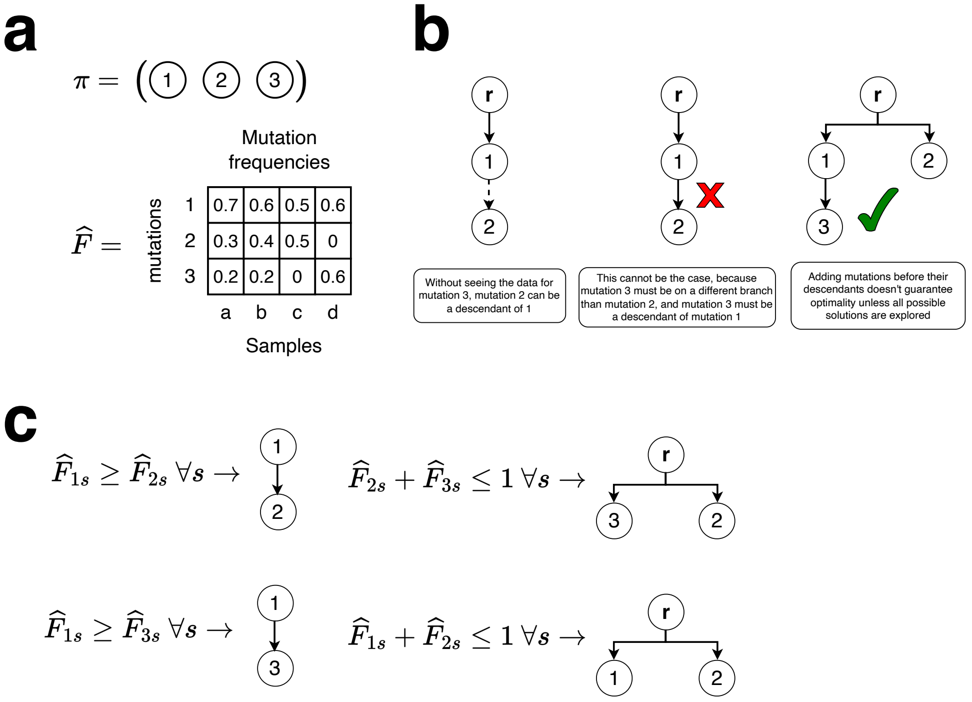

	a	b	c	d
1	0.7	0.6	0.5	0.6
2	0.3	0.4	0.5	0
3	0.2	0.2	0	0.6

$\widehat{F}_{1s} \geq \widehat{F}_{2s}\ \forall s \rightarrow$

$\widehat{F}_{2s} + \widehat{F}_{3s} \leq 1\ \forall s \rightarrow$

$\widehat{F}_{1s} \geq \widehat{F}_{3s}\ \forall s \rightarrow$

$\widehat{F}_{1s} + \widehat{F}_{2s} \leq 1\ \forall s \rightarrow$

Fig E. Counter example where $Q^{\pi}(B|D)$ may not yield the optimal tree. **a.** The mutation order π guarantees that mutations are added to the tree before their descendants. **b.** Adding mutation 2 as a descendant of 1 adheres to the ISA, but if the data for mutation 3 is observed then it must be the case that mutation 1 and 2 are on separate branches. **c.** The breakdown of the plausible pairwise evolutionary relationships for each pair of mutations, $\{(1,2),(1,3),(2,3)\}$. There is only one possible mutation tree structure implied by these pairwise relationships, and if all equally plausible solutions are not explored, $Q^{\pi}(B|D)$ may fail to recover it. This is because 2 and 3 must be on separate branches; if one were ancestral to the other, samples c and d would violate the sum constraint (Definition 2). Additionally, the only evolutionary relationship between 1 and 3 that does not violate the sum constraint is for 1 to be ancestral to 3.

A2.2.1 Greedy sampling

Let $t^{(\ell)}$ be a partial tree with a genotype matrix $B^{(\ell)}$ and log probability $\phi = \log Q^{\pi}(B^{(\ell)}|D^{(\ell)})$. The *greedy* extension of $t^{(\ell)}$ is obtained with

$$t^{\wedge(\ell+1)} = \operatorname*{arg\,max}_{i\in\{1,2,\ldots,f\}} \phi_i. \tag{25}$$

This can be applied repeatedly down the search tree to obtain a greedy sample from $Q^{\pi}(B|D)$. For many MSPP problems, there will be multiple equally likely solutions, and it is desirable to obtain some or all of these solutions. It's possible to improve this estimate by modifying Eq 25 to obtain the top-k greedy extensions:

$$t_1^{\wedge(\ell+1)}, t_2^{\wedge(\ell+1)}, \ldots, t_k^{\wedge(\ell+1)} = \arg\operatorname{top}\mathrm{k}\, \phi_i, \tag{26}$$

where arg top k selects the top-k extensions of $t^{(\ell)}$ according to their log probabilities. Repeatedly applying Eq 26 down the search tree will obtain the top-k greedy samples.

Although this approach generates multiple unique samples, it is deterministic and there are no guarantees about the optimality of these samples.

A2.2.2 Categorical Sampling

Some of the pitfalls of a greedy approach can be mitigated by *sampling* which extensions to keep. First, the set of extensions of $B^{(\ell)}$ must be treated as a categorical distribution, where each $B^{(\ell+1,i)}$ corresponds to a unique choice, and the probability it is chosen, i.e., $P(I = i)$, is proportional to its likelihood:

$$I \sim \text{Categorical}\left(\frac{\exp(\phi_i)}{\sum_{j=1}^{f} \exp(\phi_j)}, i \in \{1, 2, \ldots, f\}\right). \tag{27}$$

Sampling helps prevent the search from becoming trapped in local maxima. Running the search multiple times may yield multiple unique mutation trees, but each run samples with replacement, so this is not guaranteed. An alternative is to draw multiple samples using Eq 27 and *reject* duplicates until k unique extensions are obtained. Repeatedly applying this technique will yield of sample of k complete mutation trees without replacement from $Q^{\pi}(B|D)$. However, this approach can become problematic and expensive if there are numerous extensions or if the categorical distribution over these extensions has low entropy. In such cases, a very large number of duplicate samples might be drawn before k distinct ones are obtained [6, 7].

A2.2.3 Branch-and-bound search for the maximum probability tree

Another approach is to maximize $Q^{\pi}(B|D)$ using a branch-and-bound search. This search relies on a search tree, where internal nodes are labeled with partial mutation trees along with their (unperturbed) log probabilities, and leaves are labeled with complete mutation trees and their (unperturbed) log probabilities. In this case, the (unperturbed) log probability of an internal node serves as an upper bound for the maximum log probability of the leaves in its subtree. Although branch-and-bound will search for and find the maximum probability tree (i.e., the mode), it may involve backtracking, which requires an unbounded queue, making it impractical for large problem sizes. This is quite different from Orchard, which, instead of searching for the maximum probability tree, draws samples without replacement from $Q^{\pi}(B|D)$. Sampling from a distribution is typically easier than finding its mode, and even though Orchard doesn't explicitly search for the mode, it often finds it, particularly in highly constrained problems. Like branch-and-bound, Orchard relies on a search tree whose internal and leaf nodes are labeled with partial and complete mutation trees, but they are instead assigned perturbed log probabilities. By leveraging the Gumbel max-stability property, Orchard takes advantage of the fact that the perturbed log probability of an internal node is not just an upper bound on the perturbed log probabilities of the leaves in its subtree—it is *equal* to the maximum perturbed log probability of those leaves. Consequently, Orchard only needs to retain and then later extend those with the top-k perturbed log probabilities, allowing it to bound its queue size to k, the number of samples desired from $Q^{\pi}(B|D)$. These differences make Orchard at least as fast and more memory-efficient than a branch-and-bound search for the maximum probability tree.

A2.3 Computing U with the projection algorithm

Ideally, we would estimate the clonal proportion matrix, U, by independently optimizing each column using the following binomial objective:

$$\max_{F_{:s}, U_{:s}} \prod_{j=1}^{n} \text{Binom}(b_{js} | N_{js}, \omega_{js} F_{js}), \quad \text{subject to } \mathbf{1}^T U_{:s} \leq 1, U_{:s} \geq 0, F_{:s} = BU_{:s}.$$

However, methods that do so, e.g., rprop (Section A5.2), are typically gradient-based and can take a long time to converge. Since Orchard needs to evaluate the likelihood of numerous trees during its search, we instead use the projection algorithm [8] to quickly find a point estimate U^*. Although this algorithm was not originally designed for VAF data, which is assumed to follow a binomial sampling model, [1] demonstrated that it could be adapted for this purpose by using a Gaussian approximation to the binomial distribution. The Gaussian approximation for VAF data assumes the following:

$$b_{js} \sim \mathcal{N}(\mu_{js}, \sigma^2_{js})$$

where $\mu_{js} = N_{js}\lambda_{js}$ and $\sigma^2_{js} = N_{js}\lambda(1-\lambda)$. The definitions of μ_{js} and σ^2_{js} correspond to the mean and variance of the binomial distribution that generated the VAF data, where λ_{js} represents the true, unobserved variant allele frequency of mutation j in sample s, and $N_{js} = a_{js} + b_{js}$ is the number of reads mapped to the locus containing mutation j. We can derive a proportional Gaussian by dividing by the scalar $N_{js}\omega_{js}$:

$$\frac{b_{js}}{N_{js}\omega_{js}} \sim \mathcal{N}\left(\frac{\mu_{js}}{N_{js}\omega_{js}}, \left(\frac{\sigma_{js}}{N_{js}\omega_{js}}\right)^2\right),$$

$$\widehat{F}_{js} \sim \mathcal{N}\left(F_{js}, \frac{F_{js}}{N_{js}\omega_{js}}\left(1-\omega_{js}F_{js}\right)\right).$$

The terms $\widehat{F}_{js}$ and F_{js} are obtained using Eq 29. This Gaussian is a distribution over our observed data, which requires that we know the true variant allele frequency, λ_{js}, and mutation frequency, F_{js}. To obtain a distribution over these unknowns, we swap them with our observed values $\widehat{F}_{js}$, resulting in the following distribution:

$$F_{js} \sim \mathcal{N}\left(\widehat{F}_{js}, \frac{\widehat{F}_{js}}{N_{js}\omega_{js}}\left(1-\omega_{js}\widehat{F}_{js}\right)\right).$$

We can use this distribution to obtain a new Gaussian objective that approximates the binomial one:

$$\max_{F_{:s}, U_{:s}} \prod_{j=1}^{n} \mathcal{N}\left(F_{js} \middle| \widehat{F}_{js}, \frac{\widehat{F}_{js}}{N_{js}\omega_{js}}(1-\omega_{js}\widehat{F}_{js})\right),$$

where $\mathcal{N}(x|\mu,\sigma^2)$ is the probability density function of the Gaussian distribution. To simplify the notation, we omit the constraints. Since this objective is a product of exponentials, we can express it as follows:

$$\prod_{j=1}^{n} \mathcal{N}\left(F_{js} \middle| \widehat{F}_{js}, \frac{\widehat{F}_{js}}{N_{js}\omega_{js}}(1-\omega_{js}\widehat{F}_{js})\right) \propto \exp\left(-\sum_{j=1}^{n} - \frac{\left(F_{js}\widehat{F}_{js}\right)^2}{\sigma^2_{js}}\right),$$

where $\sigma_{js}^2 = \frac{\widehat{F}_{js}}{N_{js}\omega_{js}}(1 - \omega_{js}\widehat{F}_{js})$. As in [1], using the fact that $\exp(x)$ is a monotonic function, maximizing the Gaussian objective is equivalent to minimizing the following:

$$\min_{F_{:s}, U_{:s}} \sum_{j=1}^{n} W_{js}\left(F_{js} - \widehat{F}_{js}\right)^2, \quad \text{subject to } \mathbf{1}^T U_{:s} \le 1, U_{:s} \ge 0, F_{:s} = BU_{:s},$$

where $W_{js} = \frac{1}{\sigma_{js}^2}$. The objective function in Eq 18 decomposes into m independent problems of this form due to the squaring of the Frobenius norm.

A3 Extending a partial mutation tree

Orchard extends a partial mutation tree $t^{(\ell)}$ by adding a new mutation. There are two equivalent ways to think about the addition of a new mutation. First, we can add a new row $\mathbf{a}^{(\ell+1)}$ and column $\mathbf{d}^{(\ell+1)}$ to the corresponding genotype matrix representation of $t^{(\ell)}$, $B^{(\ell)}$, then enumerate all possible ways to populate $\mathbf{a}^{(\ell+1)}$ and $\mathbf{d}^{(\ell+1)}$ such that $B^{(\ell+1)} = \left[B^{(\ell)}, \mathbf{a}^{(\ell+1)}; \mathbf{d}^{(\ell+1)}, 1\right]$ remains a perfect-phylogeny-compatible genotype matrix. Alternatively, we can add a mutation v to $t^{(\ell)}$ by choosing parent node $u \in V^{(\ell)}$ for v, and then deciding which of u's children in $t^{(\ell)}$ become the children of v. All placements of v in $t^{(\ell)}$ can be considered by enumerating through all combinations of parents for v and which mutations become its children. As such, the number of valid ways to add v to $t^{(\ell)}$ is $\sum_{u \in V^{(\ell)}} 2^{|ch(u)|}$, where $ch(v) \subset V^{(\ell)}$ is the set of children of u in $t^{(\ell)}$. In the worst-case scenario, the mutation tree resembles a star tree where all mutations are children of the root. In such cases, the number of possible ways to add v to $t^{(\ell)}$ amounts to $2^\ell + \ell$. For example, if $u = r$ is the root which has ℓ children, then there are $\sum_{\ell'=1}^{\ell} \binom{\ell}{\ell'} = 2^\ell$ total number of placements where v is a child of u, and then it is a binary decision of whether or not v is the parent of each of the ℓ children of u. Additionally, there are ℓ other possible placements of v: it can be a child of each of the ℓ mutations in $t^{(\ell)}$, but v is not a parent of any mutations. While it's conceivable that the VAF data suggests such a tree structure, it's highly improbable if the point mutations exhibit non-zero variant allele frequencies in one or more samples. As the number of children of u with non-zero VAFs increases, the likelihood of violating the *sum constraint* (Definition 2) increases.

A3.1 Heuristic for computing the likelihood of an extension

There can be numerous extensions of $t^{(\ell)}$, many of which will have near zero probability under the approximate posterior $Q^\pi(D|B)$. Evaluating $Q^\pi(D|B)$ for each of these extensions requires computing the clonal proportion matrix, which can be very time consuming. Instead, we can quickly approximate which extensions should be kept using a special function, $H(t^{(\ell)}, f)$. This function selects f extensions of $t^{(\ell)}$ to keep, for which we can then approximate the clonal proportion matrix and properly evaluate them under $Q^\pi(D|B)$.

We'll now describe how $H(t^{(\ell)}, f)$ selects these extensions. First, we show that each placement of v into $t^{(\ell)}$ can be evaluated by how well each placements adheres to the ISA. Next, we show how to compute a probability distribution over these placements.

A3.1.1 Defining constraints on F_{vs} given the placement of v

Under the ISA, the mutation frequency matrix F has three important properties in relation to each mutation $u \in V^{(\ell)}$ for all samples $s = 1, \ldots, m$:

Definition A3.1 (F Properties)**.**

1. $0 \leq F_{us} \leq 1$
2. $F_{us} \geq \sum_{w \in ch(u)} F_{ws}$ (sum constraint)
3. $F_{us} = U_{us} + \sum_{w \in D(u)} U_{ws}$

where $D(u)$ denotes the set of all descendants of v according to the tree structure defined by $t^{(\ell)}$, and $ch(u)$ is the set of mutations that are children of u in $t^{(\ell)}$. The value U_{us} denotes the clonal proportion of mutation u in sample s which is defined as

$$U_{us} = F_{us} - \sum_{w \in ch(u)} F_{ws}, \tag{28}$$

Another important relationship we assume for F_{us} is that

$$F_{us} = \frac{\lambda_{us}}{\omega_{us}}, \tag{29}$$

where λ_{us} is the variant allele frequency of mutation u in sample s, and ω_{us} is the variant read probability of mutation u in sample s. This is equivalent to Eq 2.

All extensions of $t^{(\ell)}$ correspond to unique placements of a new mutation $v \in V \setminus V^{(\ell)}$ into the tree. These extensions can be enumerated by selecting a parent $u \in V^{(\ell)}$, and choosing which of u's children become the children of v. Once v is placed in $t^{(\ell)}$, it must also be the case that F_{vs} adheres to the constraints in Definition A3.1. We can use this fact to compute a probability distribution over all extensions of $t^{(\ell)}$ based on how well the VAF data for v supports the constrained values of F_{vs}.

Let u be the parent of v and $\chi \subseteq ch(u)$ be a set of mutations that are now the children of v. We can use Definition A3.1, Property 2, to define a set of constraints on F_{vs}. Since v is now the parent of the mutations in χ, it must be the case that:

$$F_{vs} \geq \sum_{i \in \chi} F_{is}.$$

At the same time, since v is a child of u, it must also be the case that

$$F_{us} \geq F_{vs} + \sum_{j \in ch^*(u)} F_{js},$$

where $ch^*(u) = ch(u) \setminus \chi$ is the set of mutations that remain as children of u. We can combine these two inequalities to obtain the following constraints on F_{vs}:

$$F_{us} - \sum_{j \in ch^*(u)} F_{js} \geq F_{vs} \geq \sum_{i \in \chi} F_{is}. \tag{30}$$

We can then use Equation 29 to rewrite eq 30 as

$$F_{us} - \sum_{j \in ch^*(u)} F_{js} \geq \frac{\lambda_{vs}}{\omega_{vs}} \geq \sum_{i \in \chi} F_{is},$$

$$\omega_{vs} \left(F_{us} - \sum_{j \in ch^*(u)} F_{js} \right) \geq \lambda_{vs} \geq \omega_{vs} \left(\sum_{i \in \chi} F_{is} \right), \tag{31}$$

where λ_{vs} is the VAF of mutation v in sample s and ω_{vs} is the variant read probability of mutation v in sample s. It's clear from Eq 31 that if $\chi \neq \varnothing$, then under the ISA λ_{vs} needs to fall below $\omega_{vs} \left(F_{us} - \sum_{j \in ch^*(u)} F_{js} \right)$, and above $\omega_{vs} \left(\sum_{i \in \chi} F_{is} \right)$.

Let's consider the case where v is a child of u and $\chi = \varnothing$. We can use Definition A3.1, Property 2, to derive the constraint:

$$F_{us} \geq \sum_{i \in ch(u)} F_{is} + F_{vs}.$$

By rearranging terms we obtain the following:

$$\begin{aligned} F_{us} - \sum_{i \in ch(u)} F_{is} &\geq F_{vs} \\ U_{us} &\geq F_{vs} && (32) \\ U_{us} &\geq \frac{\lambda_{vs}}{\omega_{vs}} \\ \omega_{vs} U_{us} &\geq \lambda_{vs} && (33) \end{aligned}$$

The constraint on the left hand side in Eq 32 is obtained by the definition of the clonal proportion U_{us} (Eq 28). We again use Eq 29 to obtain our final result in Eq 33.

Finally, we can use the constraints in Eq 31 and Eq 33 to define probabilities for the parent/child relationships for v:

$$p\left(u, v, \chi | \chi \neq \varnothing\right) = Pr\left[\omega_{vs}\left(F_{us} - \sum_{j \in ch^*(u)} F_{js}\right) \geq \lambda_{vs} \geq \omega_{vs}\left(\sum_{i \in \chi} F_{is}\right)\right] \quad (34)$$

$$p\left(u, v, \chi | \chi = \varnothing\right) = Pr\left[\omega_{vs} U_{vs} \geq \lambda_{vs}\right] \quad (35)$$

It's important to note that in practice Eq 34 is computed for all $\chi \in \mathcal{P}(ch(u))$, where $\mathcal{P}(ch(u))$ is the *power set*, or set of all subsets, of $ch(u)$. Note that the size of $\mathcal{P}(ch(u))$ grows exponentially with the number of children of u. Orchard has a parameter that can be set by the user which limits the number of placements scored using Eq 34. This parameter is called the *max placements* parameter, and it provides an upper limit on the number of u's children that v can parent. For example, if the *max placements* is set to 5, then $H(t^{(\ell)}, f)$ will consider all possible placements where v is a child of u, and v is the parent of any combination of i children of u, where $i = 0, \ldots, 5$. As a result, $H(t^{(\ell)}, f)$ will consider $\sum_{i=0}^{5} \binom{|ch(u)|}{i}$ possible placements of v when it's a child of u.

A3.1.2 Computing the posterior over λ_{vs}

In this section, we show how to compute the probabilities in Eq 34 and Eq 35.

Under a binomial sampling model, we assume that our observed variant allele counts b are binomially distributed according to some success probability λ, and that we've mapped a total of $N = a + b$ reads to that locus. We place no prior beliefs on the distribution of λ, and therefore use a uniform *Beta* prior on its distribution. Under these assumptions, the posterior distribution of λ for some mutation v in sample s is defined as follow:

$$\begin{aligned} b|\lambda_{vs} &\sim Binom(N_{vs}, \lambda_{vs}) \\ \lambda_{vs} &\sim Beta(\alpha_0, \beta_0) \\ \lambda_{vs}|(b = b_{vs}) &\sim Beta(\alpha_0 + b_{vs}, \beta_0 + N_{vs} - b_{vs}), \end{aligned}$$

where $N_{vs} = a_{vs} + b_{vs}$, and the *Beta prior* is uniform, so $\alpha_0 = \beta_0 = 1$. Because the *Beta* is conjugate to the the binomial, the posterior distribution of λ_{vs} is also a *Beta* distribution, in this case with $\alpha = 1 + b_{vs}$ and $\beta = 1 + a_{vs}$. We can rewrite the posterior in terms of solely λ_{vs} and X_{vs} as follows:

$$
\begin{aligned}
p(\lambda_{vs}|X_{vs}) &\propto p(X_{vs}|\lambda_{vs})p(\lambda_{vs}) \\
&\propto f(\lambda_{vs},\alpha,\beta)
\end{aligned} \tag{36}
$$

where $X_{vs} = (a_{vs}, b_{vs}, \omega_{vs})$ and $f(\lambda,\alpha,\beta)$ is the probability density function of the Beta Distribution. If we denote $y_s = \omega_{vs}\left(\sum_{i\in\chi} F_{is}\right)$ and $z_s = \omega_{vs}\left(F_{us} - \sum_{j\in ch^*(u)} F_{js}\right)$, then we can define Eq 34 as follows:

$$
\begin{aligned}
p\left(u,v,\chi|\chi \neq \varnothing\right) &= Pr\left[z_s \geq \lambda_{vs} \geq y_s\right] \\
&= \int_{y_s}^{z_s} f(\lambda_{vs},\alpha,\beta) d\lambda_{vs} \\
&= \int_{y_s}^{z_s} \frac{1}{B(\alpha,\beta)} \lambda_{vs}^{\alpha}(1-\lambda_{vs})^{\beta} d\lambda_{vs} \\
&= \frac{1}{B(\alpha,\beta)} \int_{y_s}^{z_s} \lambda_{vs}^{\alpha}(1-\lambda_{vs})^{\beta} d\lambda_{vs} \\
&= I_{z_s}(\alpha,\beta) - I_{y_s}(\alpha,\beta)
\end{aligned} \tag{37}
$$

where $I_x(\alpha,\beta)$ is the *regularized incomplete beta function*. If we let $z_s' = \omega_{vs}U_{vs}$, then we can perform a similar process to define Eq 35 as

$$
\begin{aligned}
p\left(u,v,\chi|\chi = \varnothing\right) &= Pr\left[z_s' \geq \lambda_{vs}\right] \\
&= \int_{0}^{z_s'} f(\lambda_{vs},\alpha,\beta) d\lambda_{vs} \\
&= \frac{1}{B(\alpha,\beta)} \int_{0}^{z_s'} \lambda_{vs}^{\alpha}(1-\lambda_{vs})^{\beta} d\lambda_{vs} \\
&= I_{z_s'}(\alpha,\beta)
\end{aligned} \tag{38}
$$

It's possible that y_s and z_s can be very close in Eq 37. This might occur when mutations are part of the same clone, and therefore have very close mutation frequencies across all samples. In this case, we would like Orchard to place these mutations together in the tree, however, the closeness of the mutation frequencies may make it so the ideal placements have near zero probability. To mitigate this problem, we widen the range of integration between y_s and z_s by adjusting them using binomial proportion confidence intervals. In particular, we use the Wald interval to adjust y_s and z_s:

$$
\begin{aligned}
\hat{z}_s &= z_s + \frac{2.576}{\sqrt{N_{vs}}}\sqrt{\widehat{\lambda}_{vs}(1-\widehat{\lambda}_{vs})}, \\
\hat{y}_s &= y_s - \frac{2.576}{\sqrt{N_{vs}}}\sqrt{\widehat{\lambda}_{vs}(1-\widehat{\lambda}_{vs})}.
\end{aligned}
$$

The value $\widehat{\lambda}_{vs}$ is the variant allele frequency of mutation v in sample s that's implied by the data, which can be calculated using the formula presented in Section 1.2. The value "2.576" is the target error rate that corresponds to the 99th percentile. If $y_s = z_s = \widehat{\lambda}_{vs}$, then $\hat{z}_s$ and $\hat{y}_z$ would be the 99th percentile upper confidence limit and 99th percentile lower confidence limit for $\widehat{\lambda}_{vs}$, respectively. Under these conditions the integral in Eq 37 will evaluate to nearly 1, meaning this placement agrees perfectly with the data for mutation v in sample s.

A3.1.3 Choosing a branching factor

The function $H(t^{(\ell)}, f)$ quickly evaluates all possible extensions of $t^{(\ell)}$ and returns the indices of the top-f extensions under its approximation. We can assign a "rank" to each extension using the sorted order of the likelihoods computed by $H(t^{(\ell)}, f)$: the extension with the largest likelihood is assigned rank 1, the second largest as rank 2, etc.. As Orchard builds trees, we can record these ranks and evaluate what we should set f to such that we evaluate $Q^{\pi}(B|D)$ as few times as possible, while not impacting the accuracy of Orchard's reconstructions.

We empirically determined the value of the branching factor f to use in our experiments using a validation set. We ran Orchard on this validation set with parameters that allow it to evaluate $Q^{\pi}(B|D)$ for all possible extensions by setting $f = \infty$. This does not result in a complete exhaustive search of all possible trees (which would be computationally intractable for the tree sizes in the validation set) because we limit the number of partial trees to keep track of by setting the beam width parameter k. We chose to run Orchard with two different beam widths $k = 1$ and $k = 10$, along with $f = \infty$. We decided to use 200 simulated bulk DNA cancer phylogenies originally from [9] as a validation set. These simulations mimic whole exome sequencing, with each dataset containing 100-200 mutations, 5 samples, and a read depth of 200x.

Fig F shows the percentage of ranks that led to the tree with the largest likelihood across all datasets in the validation set. Setting $k = 1$ and $k = 10$ results in very similar distributions of ranks; thus, we only show the results for $k = 1$. The 30th percentile rank is 21, the 50th percentile is 46, and the 80th percentile is 109. To find the optimal extension 80% of time, we need to evaluate $Q^{\pi}(B|D)$ for the top 100 extensions. While this is computationally tractable, in practice we found on the validation set that the results were still very good and 5x faster when considering only the top 20 extensions. Based on these findings, we chose to limit $f = 20$ in our experiments.

The results in Fig F support that $H(t^{(\ell)}, f)$ is a relatively good approximation. It's important to note that if the branching factor is smaller than the total number of possible extensions for any given partial tree, then Orchard may not find the top-k mutation trees with the largest perturbed log probabilities. However, further improvements to the heuristic could dramatically decrease run times and improve reconstructions. Additionally, it is possible to adapt this heuristic into its own MSPP reconstruction method, with the complete trees output by the method being formally evaluated using $P(B|D)$.

A3.2 Derivations

We now show that the relation in Eq 6 holds true. Let $B \in \{0,1\}_{n \times n}$ be a binary genotype matrix, where each entry $B_{ij} \in \{0,1\}$ is a binary random. We denote the set of all of these binary random variables with $A_B = \{B_{ij} | B_{ij} \text{ is an element of } B\}$. The joint posterior over this set is equivalent to Eq 3:

$$P(B|D) = P(A_B|D)$$

If $A_{B_{:j}} = \{B_{1j}, B_{2j}, \ldots, B_{nj}\}$ is the set of binary random variables associated with the j-th column of B, then the joint posterior can be rewritten as:

$$P(A_B|D) = P(A_{B_{:1}}, A_{B_{:2}}, \ldots, A_{B_{:n}}|D) = \prod_{j=1}^{n-1} P(A_{B_{:j+1}}|A_{B_{:1}}, \ldots, A_{B_{:j}}, D),$$

which is a straight forward application of the chain rule of probability. We can perform a similar factorization using arbitrary subsets of A_B. Consider $B^{(\ell+1)} = \left[B^{(\ell)}, \mathbf{a}^{(\ell+1)}; \mathbf{d}^{(\ell+1)}, B_{\ell+1,\ell+1}\right]$, where:

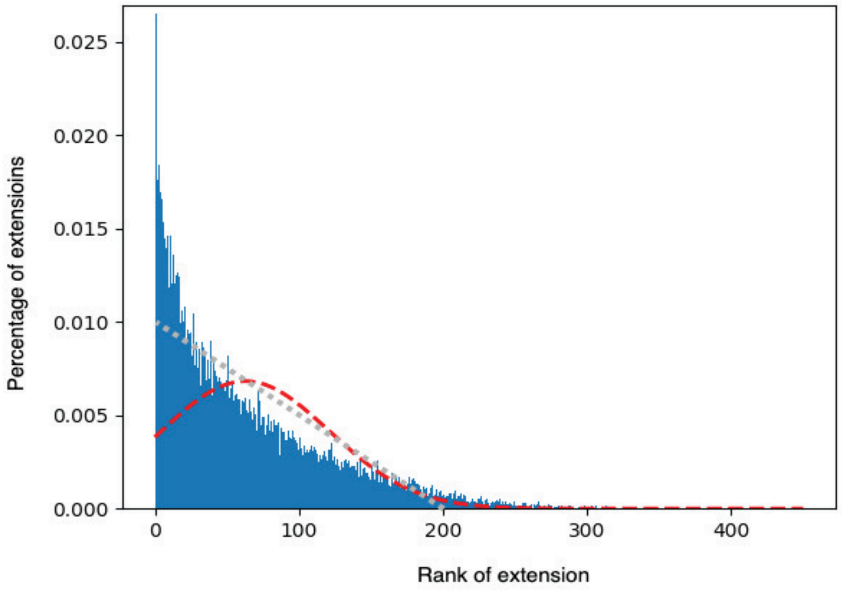

Fig F. The percentage of extensions of each rank that led to the tree with the largest likelihood across all data sets in the validation set. The validation set consisted of 200 simulated cancers originally from [9]. The grey dotted line represents the percentage of extensions that we would expect for each rank if they were randomly chosen, assuming only a linear tree structure. The red dotted line is a Gaussian distribution fit to the rank data.

- $\mathbf{a}^{(\ell+1)}$ represents the first ℓ elements of the $(\ell+1)$-th column of B
- $\mathbf{d}^{(\ell+1)}$ represents the first ℓ elements of the $(\ell+1)$-th row of B
- $B^{(\ell)}$ is a submatrix containing the first ℓ rows and columns of B
- $B_{\ell+1,\ell+1}$ is the $(\ell+1)$-th entry on the diagonal of B.

Let $A_{\mathbf{a}^{(\ell+1)}}, A_{\mathbf{d}^{(\ell+1)}}, A_{B^{(\ell)}}, A_{B_{\ell+1,\ell+1}}$ denote the sets of binary random variables corresponds to these parts of B, then we can rewrite the factorization again as:

$$P(B|D) = P(A_B|D) = \prod_{\ell=1}^{n-1} P(A_{\mathbf{a}^{(\ell+1)}}, A_{\mathbf{d}^{(\ell+1)}}, A_{B_{\ell+1,\ell+1}}|A_{B^{(\ell)}}, D) = \prod_{\ell=1}^{n-1} P(B^{(\ell+1)}|B^{(\ell)}, D),$$

which shows that Eq 6 holds true.

A4 Phylogeny-aware clustering

The "phylogeny-aware" clustering algorithm we propose consists of two steps: (1) agglomerative clustering, and (2) model selection. We will now describe these steps in detail.

A4.1 Agglomerative clustering

The phylogeny-aware clustering algorithm we propose is an *agglomerative clustering algorithm*. Agglomerative clustering, also known as *hierarchical agglomerative clustering*, is a greedy clustering method that iteratively merges pairs of nodes in a graph until the graph consists of only a single node [10]. Generally, agglomerative clustering methods have a worst case time complexity of $O(n^3)$ [10]. In order to determine which pair of nodes should be combined at each step during agglomerative clustering, a *cluster linkage* criterion is used to evaluate the dissimilarity between all adjacent nodes in the graph. Since adjacent nodes u and v contain one or more objects (mutations), the cluster linkage criterion is generally a function of the dissimilarity between each pair of objects (mutations) (i, j) where $i \in u, j \in v$. The phylogeny-aware clustering algorithm iteratively joins nodes based on minimizing the linkage criterion:

$$\min_{u,v} d(u, v),$$

where $d(u, v)$ is a distance function. In our case, we chose to use Ward's method for the linkage criterion because it is a standard for agglomerative clustering algorithms:

$$d(u, v) = \frac{n_u n_v}{n_u + n_v} \|\bar{F}_u - \bar{F}_v\|^2,$$

where n_u is the number of mutations associated with node u, $\bar{F}_u$ is the average mutation frequency of the mutations contained in node u, and $\|\cdot\|$ is the Euclidean norm.

The phylogeny-aware clustering algorithm performs agglomerative clustering on a directed rooted tree, and so special care must be taken when merging nodes. Importantly, we at first only consider joining adjacent nodes that are on the same *linear segment* until all such pairs are exhausted. We define a linear segment as a linear sequence of nodes, where each node has exactly 1 or 0 child nodes. Nodes that are not on the same linear segment are distinguished by a *speciation* or *branching* event, representing the occurrence of distinct clones. Mutations that occur after a branching event are unique to that clone and all its subclones. However, we relax this constraint and allow the algorithm to consider merges that either join distinct branches or join a child node to a parent that has more than one child once there are no more linear segments. Thus, the algorithm tries to adhere to the phylogenetic constraints when clustering until no merges can be performed without violating them. This approach allows the user to consider clone trees containing anywhere from 1 to n clones.

At each iteration, the phylogeny-aware clustering algorithm merges two adjacent nodes. Consequently, at iteration i, the tree consists of $n - i$ clones. If we restrict the phylogeny-aware clustering algorithm to only merge nodes on linear segments, then the minimum number of clones resolvable in a mutation tree is defined by the number of linear segments, N_{seg}. Therefore, if N_{seg} is the number of linear segments in a mutation tree and n is the number of mutations, then there are at most $n - N_{\text{seg}}$ clones trees that can be resolved with agglomerative clustering without violating phylogenetic constraints. The phylogenetic constraints that should be adhered to when performing agglomerative clustering on a tree are illustrated in Figure Ga. The concept of a linear segment, calculating the minimum number of clones in a tree, and calculating the maximum number of clone trees resolvable without violating phylogenetic constraints are illustrated in Figure Gb.

A4.2 Estimating the cluster variant allele frequency

The likelihood of each clustering with $c = 1, \ldots, n$ clones is calculated using Eq 23. In order to evaluate the likelihood, the variant allele frequency for each clone i in sample s,

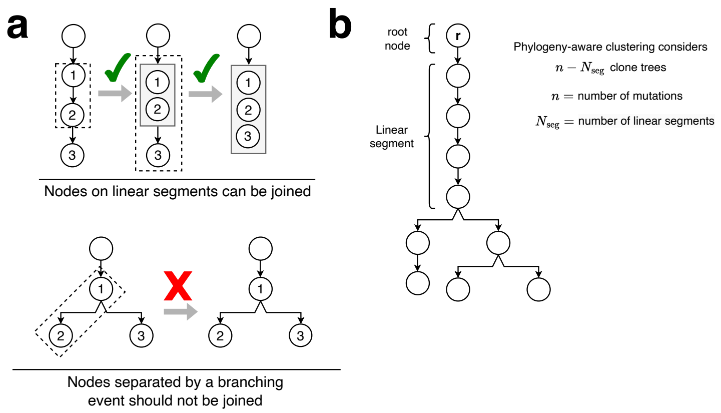

Fig G. Overview of how agglomerative clustering ideally operates on a tree. **a.** Phylogenetic constraints imply that only adjacent nodes on the same linear segment should be considered for merging. Merging nodes that are separated by a branching event violates the phylogenetic constraints. Dotted rectangles enclose the nodes considered for joining. **b.** The number of clone trees that can be resolved without violating phylogenetic constraints can be calculated using the formula: $n - N_{\text{seg}}$, where n is the number of mutations in the mutation tree, and N_{seg} is the number of linear segments in the tree. For the tree shown in **b**, $n = 9$, and $N_{\text{seg}} = 5$, therefore, there are 4 clones trees that can be resolved with agglomerative clustering if phylogenetic constraints aren't violated.

λ_{is}, needs to be estimated. The mutation frequencies, F, and the VAF data, D, are used estimate λ_{is} as follows:

$$\lambda_{is} = \frac{\sum_{j|Z_{ji}=1} F_{js}\omega_{js}N_{js}}{\sum_{j|Z_{ji}=1} N_{js}}. \tag{39}$$

The quantity $F_{js}\omega_{js}N_{js}$ estimates the number of variants reads expected to be observed in sample s given that $\lambda_{js} = F_{js}\omega_{js}$ and a total of N_{js} reads were mapped to the locus containing mutation j. The numerator in Eq 39 is the sum of the expected number of variant reads in sample s for each mutation assigned to clone i, while the denominator is the total number of reads mapped to each of the loci containing a mutation assigned to clone i. The relationship $\lambda_{js} = F_{js}\omega_{js}$ follows directly from Eq 2.

A4.3 Model selection for phylogeny-aware clustering

The phylogeny-aware clustering method outputs n different models as a result of its agglomerative clustering scheme. Each model contains a unique number of clusters from 1 to n. We can then either manually select the number of clusters believed to be in the data set, or we can use a model selection criterion to choose the number of clusters. One of the most well known model selection criteria is the Bayesian Information Criterion (BIC) [11]. The BIC chooses a model from a finite set of proposed models based on a penalized likelihood of the data given the model and its parameters. The BIC penalizes large models in attempt to select a model that performs well on the data without overfitting [11]. The BIC is formally defined as

$$\text{BIC} = klog(n) - 2\log(L(D|\theta)), \tag{40}$$

where $L(D|\theta)$ is the likelihood of the data D given the parameters θ, k is the number of parameters in θ, and n are the number of data points in D. Generally, the model with

the smallest BIC is chosen.

The model selection problem is well studied, and many different model selection criteria have been proposed. One generalization of the BIC is the Generalized Information Criterion (GIC) [12]. The GIC introduces a parameter λ which is a general penalty term:

$$GIC = k\lambda - 2log(L(D|\theta)). \tag{41}$$

The BIC and the GIC are equivalent when $\lambda = log(n)$. Generally, λ is used to change the penalty based on the size of the data set and its dimensionality. For higher-dimensional data, one choice of λ that has been previously used is $\lambda = log(m)log(n)$, where m is the number of features in the data [12]. To choose a model output the phylogeny-aware clustering algorithm, we use the GIC with $\lambda = log(m)log(n)$, which heavily penalizes larger models when the bulk data contains many samples.

A5 Evaluation metrics

In this section, we describe the evaluation metrics used to score mutation trees, and mutation clusters.

A5.1 Perplexity

We use the perplexity to evaluate a mutation tree based on its mutation frequency matrix F. Assuming we've sampled I total unique trees $A = \{t_1, t_2, \ldots, t_I\}$ with some probability distribution over this set of trees $0 \le p(t_i) \le 1$ with $\sum_{i=1}^{I} p(t_i) = 1$, we can compute the perplexity of this set of trees, $PP(A)$, as

$$PP(A) = 2^{\epsilon} \tag{42}$$

$$\epsilon = -\frac{1}{nm}\sum_{j=1}^{n}\sum_{s=1}^{m} log_2\left(\sum_{i=1}^{I} p\left(X_{js}|F_{js}^{(i)}\right)p(t_i)\right) \tag{43}$$

where $F^{(i)}$ is the mutation frequency matrix for i-th tree, and $p\left(X_{js}|F_{js}^{(i)}\right) = \text{Binom}(b_{js}|b_{js} + a_{js}, \omega_{js}F_{js}^{(i)})$. We choose the following definition for $p(t_i)$:

$$p(t_i) = \frac{\log P(D|B^{(i)}, U^{(i)})}{\sum_{i'=1}^{I} \log P(D|B^{(i')}, U^{(i')})}, \tag{44}$$

where $B^{(i)}$ and $U^{(i)}$ are the binary genotype matrix and clonal proportion matrix for the i-th tree, respectively. The numerator and denominator in this equation are computed using Eq 22. Using this definition means that Eq 42 is a likelihood weighted average perplexity of the mutation frequency matrices $\{F^{(i)} : i = 1, \ldots, I\}$ under a binomial sampling model.

We can measure the perplexity of a mutation frequency matrix reconstructed by a method in reference to a baseline perplexity using the ratio $\frac{2^{\epsilon_\Omega}}{2^{\epsilon_{\text{base}}}}$, where ϵ_Ω is equivalent to Equation 43 and ϵ_{base} is the exponent for the baseline perplexity being compared against. The perplexity can sometimes be very large; therefore, we choose to transform the perplexity and the perplexity ratio using a base 2 logarithm, resulting in the *log perplexity* and *log perplexity ratio*. The log perplexity ratio has also been called the *VAF Reconstruction loss* [1]. We denote the log perplexity ratio as $\hat{\epsilon}$, where $\hat{\epsilon} = log_2\left(\frac{2^{\epsilon_\Omega}}{2^{\epsilon_{\text{base}}}}\right) = \epsilon_\Omega - \epsilon_{\text{base}}$. Therefore, it is possible that $\hat{\epsilon}$ is negative showing that the

mutation frequency matrix F for the tree(s) reconstructed by a method fit the VAF data better than the baseline mutation frequency matrix. With simulated data, the baseline is the ground-truth mutation frequency matrix $F^{(\text{true})}$ used to generate the simulated VAF data. With real data, the baseline is a *maximum a posteriori* (MAP) mutation frequency matrix $F^{(\text{MAP})}$ fit to the expert derived tree. The MAP mutation frequency matrices fit to the expert-derived trees were calculated using a gradient based method called *resilient backpropagation* (rprop) [1], which is described in the next section.

A5.2 Computing the *maximum a posteriori* mutation frequency matrix for expert-derived trees

A bulk DNA clone tree derived by experts may not have a mutation frequency matrix that adheres to a perfect phylogeny. However, we can obtain a very close approximation of its mutation frequency matrix by fitting a maximum a posteriori (MAP) estimate of F after observing the expert-derived tree. Although Orchard uses the projection algorithm [8] to fit the mutation frequency matrix F, this algorithm optimizes a Gaussian approximation to the binomial likelihood, so it is not guaranteed to find the maximum likelihood estimate. However, a very close approximation to the maximum likelihood of F can be obtained using gradient-based methods. Unfortunately, these methods take a long time to converge, making them impractical for large reconstruction problems or in situations where many F matrices need to be estimated. However, gradient-based methods are quite useful for fitting a MAP estimate of F given a clone tree derived by experts. One such gradient-based method used for this task in prior research is rprop, or *resilient backpropagation* [1]. We chose to use rprop to find the MAP mutation frequency matrix, $F^{(\text{MAP})}$, for the B-ALL expert-derived clone trees. We ran rprop for 30,000 epochs on each B-ALL data set to estimate $F^{(\text{MAP})}$ for each expert-derived clone tree.

A5.3 Relationship reconstruction loss

Given a mutation tree $t = \{V, E, M\}$ and an ordered pair of nodes $(u, v) \in V$, there are 3 possible pairwise evolutionary relationships the ordered pair (u, v) can have:

Definition A5.1 (Pairwise Evolutionary Relationships)**.**

Ancestral: u is an ancestor of v, i.e., v contains the mutation(s) associated with u, but v contains one or more mutations not present in u.

Descendant: v is an ancestor of u, i.e., the same as above but u and v are switched.

Branched: u and v share some common ancestor, but neither u nor v are ancestral to each other.

We denote the 3 possible pairwise evolutionary relationships from Definition A5.1 for the ordered pair of nodes (u, v) as one of the following:

$$R_{uv} = \text{ ancestral}$$
$$R_{uv} = \text{ descendant}$$
$$R_{uv} = \text{ branched},$$

where R_{uv} denotes the evolutionary relationship between the ordered pair (u, v). Note that R_{uv} can be derived from the binary genotype matrix B associated with a tree t as

follows:

$$R_{uv} = \begin{cases} \text{ancestral} & \text{if } B_{uv} = 1, B_{vu} = 0 \\ \text{descendant} & \text{if } B_{uv} = 0, B_{vu} = 1 \\ \text{branched} & \text{if } B_{uv} = B_{vu} = 0 \end{cases}$$

and if $B_{uv} = B_{vu} = 1$ then B is not consistent with any tree, as this implies a cycle.

The *relationship reconstruction loss* measures how well the pairwise relationships between mutations in some proposed tree t match the pairwise relationships in a set of N ground truth trees:

$$A^{(\text{true})} = \left\{ t_1^{(\text{true})}, t_2^{(\text{true})}, \ldots, t_N^{(\text{true})} \right\}.$$

We primarily use the relationship reconstruction loss when evaluating reconstructions for simulated mutation tree reconstruction problems. We generated simulated trees and VAF data using the Pearsim software (https://github.com/morrislab/pearsim). The Pearsim software starts with a ground truth mutation frequency matrix $F^{(\text{true})}$ and enumerates all possible trees that fit $F^{(\text{true})}$ without violating the ISA. This process results in a set $A^{(\text{true})}$. Since each pair of nodes (u, v) in a tree t can have one of the three mutually exclusive pairwise relationships (see Definition A5.1), we define the probability that (u, v) has a particular pairwise evolutionary relationship in t as

$$p(R_{uv} = e|t) = \begin{cases} 1 & \text{iff } R_{uv} = e \text{ in } t \\ 0 & \text{otherwise,} \end{cases}$$

where $e \in \{\text{ancestral}, \text{descendant}, \text{branched}\}$ as defined in Definition A5.1, and R_{uv} is the pairwise evolutionary relationship between nodes u and v defined by the tree t.

Let $A = \{t_1, t_2, \ldots, t_I\}$ be the set of I unique trees reconstructed by a MSPP method. We can compute the probability that a particular evolutionary relationship e occurs between nodes u and v in this set as

$$p(R_{uv} = e|A) = \sum_{i=1}^{I} p(R_{uv} = e|t_i) p(t_i). \tag{45}$$

We use the same definition for $p(t_i)$ from Eq 44. We can compute the same probability with the set of N true trees, only we use a uniform prior $p(t^{(\text{true})}) = \frac{1}{N}$. We denote the posterior distribution over the pairwise relationships in our set of I proposed trees as $p(R_{uv}|A)$, while we denote the posterior distribution over the pairwise relationships in our set of N true tree as $p(\tilde{R}_{uv}|A^{(\text{true})})$. We measure the difference between these two distributions using the Jensen-Shannon divergence (JSD) while normalizing over the total number of pairs of mutations in the tree

$$\epsilon_R = \frac{2}{n(n+1)} \sum_{u,v} JSD(R_{uv} || \tilde{R}_{uv}) \tag{46}$$

A5.4 Adjusted Rand Index

The Rand Index is used to compare two clusterings based on the pairs of elements that co-occur (or do not co-occur) in the same cluster. The adjusted Rand Index is a normalized version of the Rand Index. The Rand Index is a value between 0 and 1, while the adjusted Rand Index is a value between -1 and 1, where a value below 0 is obtained if the difference between the two clusterings is worse than what would be expected if the clusterings were randomly generated. For a bulk data set containing n mutations, there are $\binom{n}{2}$ possible pairs of mutations, denoted by the set A. The set P represents the pairs of mutations that co-occur in the same clusters as determined by a

mutation clustering algorithm. The set T represents all pairs of mutations that co-occur in the baseline clusters, such as those from ground truth or expert-derived data. We use these sets to define the following:

- $TP = |P \cap T|$ is the number of *true positives.*
- $FP = |P \setminus T|$ is the number of *false positives.*
- $FN = |T \setminus P|$ is the number of *false negatives.*
- $TN = |(A \setminus P) \cap (A \setminus T)|$ is the number of *true negatives.*

The Rand Index can be defined in terms of the number of pairs of mutations that are classified as true positives (TP), false positives (FP), false negatives (FN), and true negatives (TN). We compute the Rand Index as

$$RI = \frac{TP + TN}{TP + FP + FN + TN}, \tag{47}$$

and we can compute the adjusted Rand Index as

$$ARI = \frac{2(TP \times TN - FN \times FP)}{((TP + FN) \times (FN + TN) + (TP + FP) \times (FP + TN))}. \tag{48}$$

A6 Additional Experiments

A6.1 Benchmarking on 576 simulated clone tree data sets

We also evaluated Orchard, Pairtree, and CALDER on 576 simulated clone tree data sets originally from [1]. These clone tree data sets were simulated using the *Pearsim* software (https://github.com/morrislab/pearsim). Each of the 576 simulated clone tree data sets had varying numbers of subclones (3, 10, 30, 100), average mutations per subclone (10, 20, 100), cancer samples (1, 3, 10, 30, 100), and sequencing depths (50x, 200x, 1000x).

Fig H shows the performance of Orchard, CALDER, and Pairtree on the 576 simulated clone tree data sets. These results generally reiterate the findings described in Section 1.8. Orchard and Pairtree were able to succeed on all 576 simulated clone tree data sets. CALDER succeeded on 51% (92/180) of 3-clone data sets, 30% (54/180) of 10-clone data sets, 34% (37/108) of 30-clone data sets, and 6% (6/108) of 100-clone data sets. On the 3-clone, 10-clone, and 30-clone data sets, Orchard and Pairtree had similar performance for log perplexity ratio, and relationship reconstruction loss. Orchard in both of its configurations ($k = 1$ and $k = 10$) had faster run times than Pairtree on all data sets with 30 or fewer clones. On datasets with 100 clones, CALDER outperformed Pairtree in both log perplexity ratio and relationship reconstruction loss on the subset of datasets where it was successful. Meanwhile, Orchard far outperformed both CALDER and Pairtree on all 100-clone data sets for both metrics. Orchard ($k = 1$) was between 5-100x faster than both CALDER and Pairtree on 100-clone data sets, and Orchard ($k = 10$) had similar run times to Pairtree on the 100-clone data sets.

One notable result in Fig H is that CALDER has more successes on the 30-clone datasets compared to the 10-clone datasets. We believe this phenomenon is primarily due to CALDER's optimization routine, which at times discards data if certain constraints are violated. Although the Gurobi optimizer (https://www.gurobi.com/) CALDER uses to solve its mixed integer linear program (MILP) problem formulation is deterministic, the solution path it takes is machine dependent since libraries and other utilities may vary between different machines. Therefore, it's possible for CALDER to

discard different data during separate runs on separate machines, since the solution path may be different. This may explain why we get more failures on 10-clone data sets compared to 30-clone data sets, and more generally, why the results shown here are different than those shown in [1].

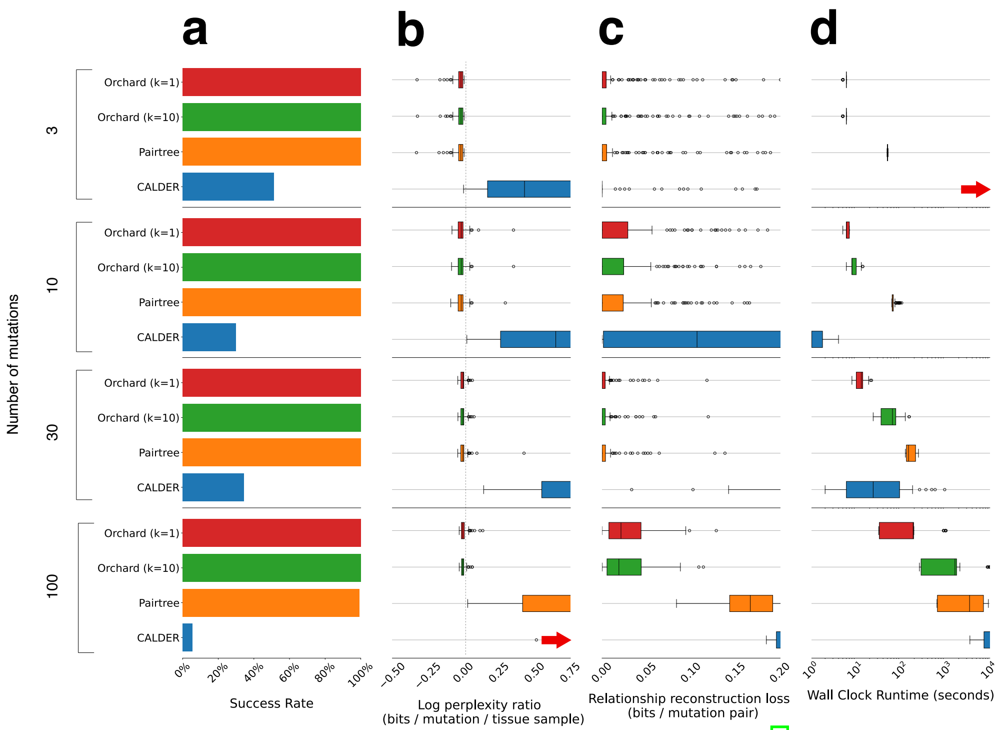

Fig H. Benchmark performance on 576 simulated data sets from [1]. The simulation results are grouped by the number of subclones (rows), and these groups are referred to as a *problem size*. **a.** Bar plots show the success rate for each method on a problem size. A method is successful on a reconstruction problem if it produces at least one valid tree. The distributions in b-d only reflect data sets where a method was successful. **b.** Box plots show the distribution of log perplexity ratio for each method on a problem size. Log perplexity ratio is reported relative to the true mutation frequency matrix $F^{(\text{true})}$ used to generate the VAF data, and therefore can be negative. A red arrow means the results for the method on a problem size occur beyond the x-axis limit. **c.** The distribution of relationship reconstruction loss for each method on a problem size. **d.** The distributions of wall clock run time in seconds.

A6.2 Comparing Orchard with greedy sampling on 90 simulated cancers

An alternative approach to sampling from $Q^{\pi}(B|D)$ is “greedy” sampling, as described in Section A2.2.1. Fig I compares Orchard versus Greedy sampling across the 90 simulated mutation tree data sets. The results in Fig Ia and Ib indicate that Orchard’s sampling approach reduces the variance of the log perplexity ratio and improves relationship reconstruction loss, resulting in slightly better reconstructions compared to a greedy search strategy. Fig Ic demonstrates that Greedy sampling generally outperforms Orchard in terms of speed for larger mutation tree reconstruction problems (100+ mutations). These findings suggest that while Greedy sampling’s runtime scales

more favorably for larger problems, it may sacrifice reconstruction quality.

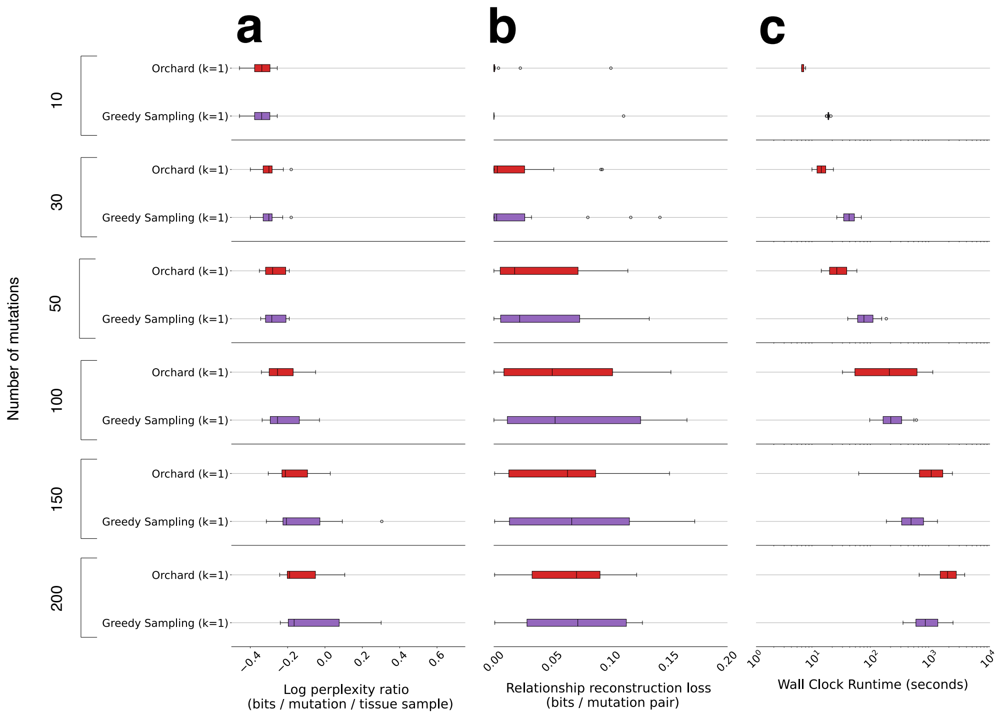

Fig I. Comparison of Orchard versus Greedy sampling on the 90 simulated mutation tree data sets. The simulation results are grouped by the number of mutations (rows), and these groups are referred to as a *problem size*. These results show that Orchard generally produces better reconstructions compared to Greedy sampling, with the caveat of slightly longer run times. **a.** The distribution of log perplexity ratio for each method on a problem size. Log perplexity ratio is reported relative to the true cellular prevalence matrix $F^{(\text{true})}$ used to generate the data, and therefore can be negative. **b.** The distribution of relationship reconstruction loss for each method on a problem size. **c.** The distributions of wall clock run time in seconds.

A6.3 Comparing Orchard using the $\widehat{F}$-sum versus randomized mutation order

The accuracy of $Q^{\pi}(B|D)$ depends on the mutation order π. In Section A2.1, we introduced the $\widehat{F}$-sum mutation order and discussed why it can improve the accuracy of $Q^{\pi}(B|D)$. Here, we run Orchard with both the $\widehat{F}$-sum ordering and a randomized order, and evaluate their impact on Orchard's ability to sample from $Q^{\pi}(B|D)$.

Orchard was run with the parameters $k = 1$ and $f = 20$. We generated randomized mutation orders by randomly permuting π. We denote Orchard run with the randomized order as "Orchard (k=1, randomized)". In Fig J, we compare Orchard's reconstructions using the $\widehat{F}$-sum and randomized mutation orders on the 90 simulated mutation tree data sets. As shown in Fig Ja and Jb, randomizing the mutation order results in a slightly higher log perplexity ratio and relationship reconstruction loss compared to the $\widehat{F}$-sum order, indicating worse reconstructions. Additionally, Fig Jc shows that randomizing the mutation order leads to slightly longer run times. We believe this increase in run times and the worse reconstructions are related; poor

mutation placements force the projection algorithm [8] to adjust more values in U to adhere to the perfect phylogeny constraints, resulting in increased run times.

Although randomizing the mutation order results in slightly worse reconstructions, it's evident that $Q^{\pi}(B|D)$ remains an accurate approximation regardless of the setting of π. These results also support that the heuristic $H(t^{(\ell)}, f)$ described in Section A3.1 is robust to any mutation order.

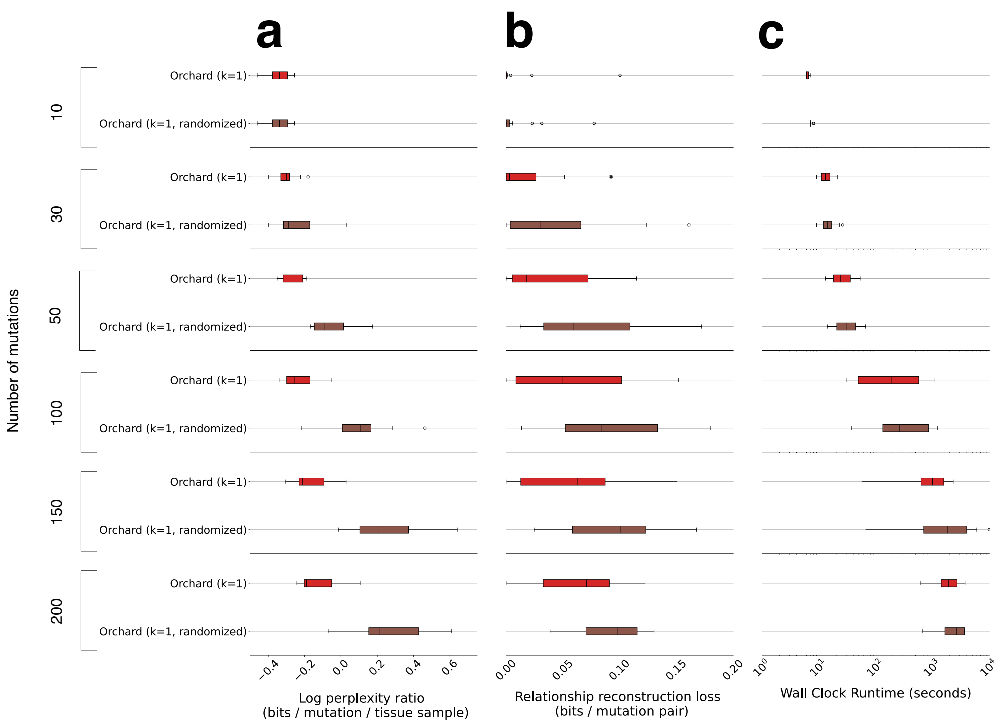

Fig J. Comparison of Orchard's performance with a randomized mutation order versus the $\widehat{F}$-sum order on 90 simulated mutation tree data sets. The simulation results are grouped by the number of mutations (rows), and these groups are referred to as a *problem size*. These results show that using a randomized order can result in slightly worse reconstructions compared to using the $\widehat{F}$ sum order described in Section A2.1. **a.** The distribution of log perplexity ratio for each method on a problem size. Log perplexity ratio is reported relative to the true cellular prevalence matrix $F^{(\text{true})}$ used to generate the VAF data, and therefore can be negative. **b.** The distribution of relationship reconstruction loss for each method on a problem size. **c.** The distributions of wall clock run time in seconds.

A6.4 Evaluating Orchard on 1000-node mutation trees

To evaluate Orchard's ability to reconstruct extremely large mutation trees, we selected four datasets from the 576 simulated clone trees used in Section A6.1, each containing 1,000 mutations. These data sets had 1,000 mutations, 10 samples, and a read depth of 50x.

Orchard completed each reconstruction in approximately 26 ± 0.5 hours. These reconstructions closely matched the data fit of the ground truth mutation frequency matrix $F^{(\text{true})}$ used to generate the simulated VAF data, achieving an average log perplexity ratio of 0.025 across the four datasets. However, the pairwise relationships

recovered by these trees were somewhat crude, with an average relationship reconstruction loss of 0.33. These results are expected, considering that a read depth of 50x may not be adequate for the precise resolution of all pairwise relationships. However, they demonstrate that Orchard can still identify trees with a good fit to the data despite this limitation.

A6.5 Mutation tree reconstructions for the 14 B-ALL data sets

In this section, we evaluate mutation tree reconstructions for the B-ALL data. These 14 data sets contain between 16 and 292 mutations, with a median of 40, resulting in very large mutation trees. Due to CALDER's high failure rate on data sets with more than 30 mutations (see Fig 3), it was excluded from this analysis. As we do not have expert-derived mutation trees for the 14 B-ALL data sets, we use the MAP mutation frequency matrix $F^{(\mathrm{MAP})}$ for each data set's expert-derived clone tree as a baseline. Fig Ka and Kb shows the log perplexity ratio for Orchard ($k = 10$) and Pairtree on each B-ALL data set. It illustrates that while Orchard matches the performance of Pairtree on reconstruction problems with fewer than 50 mutations, it significantly outperforms Pairtree on problems with more than 50 mutations. Notably, on mutation tree reconstruction problems with 129 mutations (SJETV010, Fig Ka) and 292 mutations (SJBALL022610, Fig Kb), Orchard beats Pairtree by more than 3 and 25 bits, respectively. The results in Fig Ka and Kb also illustrates that the mutation trees reconstructed by Orchard consistently exhibit better agreement with the VAF data compared to the expert-derived clone trees. For three particular cases (SJVET047, SJETV010, SJBALL0222610), the mutation trees reconstructed by Orchard have a relative decrease in perplexity that is greater than 1 bit, suggesting the possibility that the expert-derived mutation clusters for these patients are incorrectly grouping distinct subclones together.

A6.6 Additional experiment for SJBALL022611

In Fig 4a, we showed that providing more samples to Pairtree diminished its reconstruction accuracy, while Orchard's reconstructions were accurate regardless of the number of samples used. In this section, we extend this experiment by randomly selecting samples for each sample size (2, 3, 5, 10, 15, 20, 25) and evaluating Orchard's and Pairtree's reconstructions on these random subsets. For each sample size, five random subsets were chosen. Fig L shows the mean and 95% bootstrap confidence intervals for each sample size across the five trials, which are consistent with the results in Fig 4a.

A6.7 Evaluating the phylogeny-aware clustering algorithm on 14 B-ALL data sets

In this section, we compare the mutation clusters inferred by the "phylogeny-aware" clustering algorithm to those inferred by VAF based clustering methods on the 14 B-ALL data sets. The phylogeny-aware clustering algorithm was provided the best mutation tree reconstructed by Orchard and Pairtree for each B-ALL data set, corresponding to those in Section A6.5. We then use three state-of-the-art VAF-based mutation clustering methods on each data set: PyClone-VI [13], VIBER [14], and SciClone [15]. We compare the subclones output by each method to the expert-defined clusters using the *Adjusted Rand Index* (ARI). The ARI is a measurement of the percentage of correctly co-clustered mutations adjusted for randomness [16].

Fig M compares the distributions of the Adjusted Rand Index for each method across the 14 B-ALL data sets. We use "Orchard+PAC" and "Pairtree+PAC" to refer

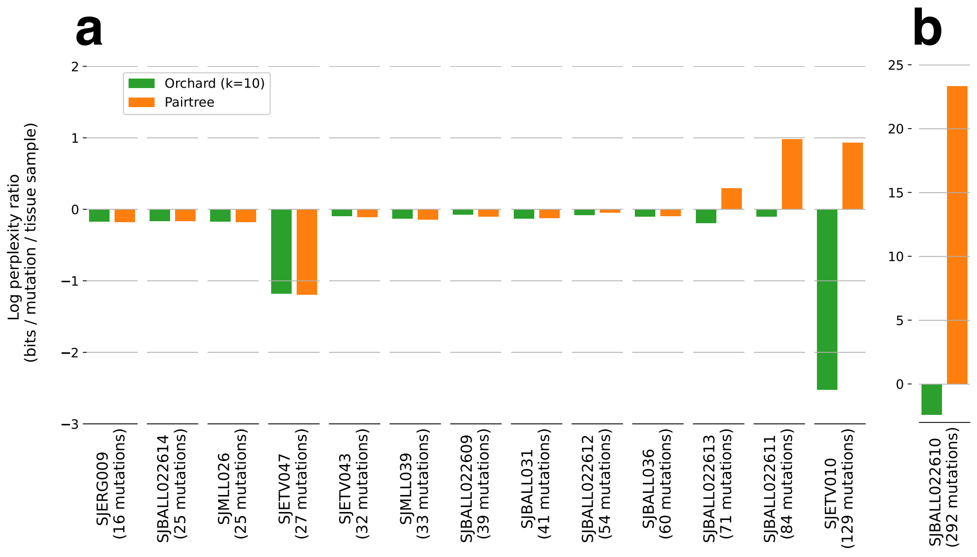

Fig K. Evaluation of mutation tree reconstructions for the 14 B-ALL data sets. Log perplexity ratio is reported relative to the maximum a posteriori (MAP) mutation frequencies $F^{(\mathrm{MAP})}$ for the expert derived clone trees. **a-b.** Bar plots show the log perplexity ratio for the mutation trees reconstructed by Orchard ($k = 10$) and Pairtree using the mutation data for each B-ALL data set. Part **b** has a larger y-axis range to accommodate Pairtree's large log perplexity ratio on the 292 node data set.

to the phylogeny-aware clustering method applied to mutation trees constructed by Orchard and Pairtree, respectively. Mutation clusters inferred by "Orchard+PAC" were most similar to the expert-derived mutation clusters for the majority of data sets. The breakdown of each method's performance on the individual data sets are shown in Table B. While the phylogeny-aware clustering method matched or outperformed all other clustering methods across the majority of B-ALL data sets, it fell short in recovering mutation clusters competitive with state-of-the-art VAF-based clustering methods specifically for SJBALL022610. In fact, for SJBALL022610, the clusters inferred by the phylogeny-aware method using Orchard's tree were poorer than those inferred using Pairtree's tree, despite Orchard's reconstructed tree being significantly better than Pairtree's (see Fig K). This could be due to the algorithm's clustering model being too simplistic for handling such a large data set with 292 mutations detected across 27 samples. It employs a non-probabilistic merging strategy to identify clones in mutation trees, with additional phylogenetic constraints that dictate when nodes can be merged. It could also be the case that the trees reconstructed by both Pairtree and Orchard for SJBALL022610 are too crude to accurately recover clones.

A6.8 Comparing expert-derived trees to those inferred using "phylogeny-aware" clustering

In this section, we use the mutation clusters from Section A4 to construct clone trees. Note that trees with more clones tend to have a better data fit because the clonal frequencies can align more closely to fewer mutations. It's important to consider this when comparing results between methods that output clone trees of different sizes. To evaluate our new clonal tree reconstruction approach against the classical approach, which we introduced in Section 1.9, we reconstructed clonal trees for the 14 B-ALL data

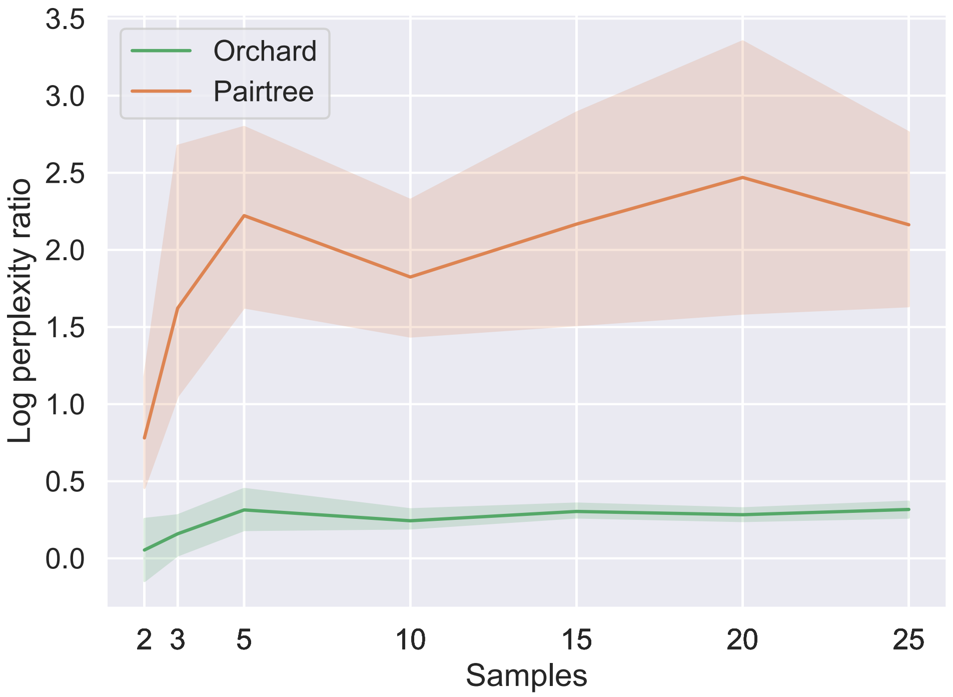

Fig L. Line plot of the log perplexity ratios for trees reconstructed by Orchard and Pairtree for SJBALL022611, shown as a function of the number of samples. For each sample size, five trials were conducted, with samples randomly selected from the 29 available samples of SJBALL022611. The dark line represents the mean log perplexity ratio across trials for each method, while the shaded area indicates the 95% bootstrap confidence interval around the mean.

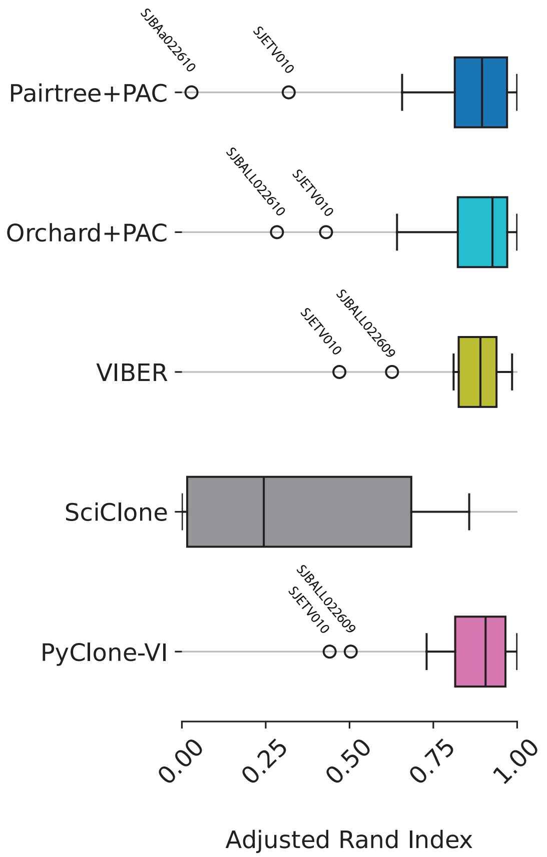

Fig M. Box plots of the Adjusted Rand Index (ARI) comparing the "phylogeny-aware" clustering method with state-of-the-art VAF-based mutation clustering methods across 14 B-ALL data sets. Adjusted Rand Index is measured in reference to the expert-derived mutation clusters for the 14 B-ALLs. Outliers are labeled by their unique ID.

set using the following methods:

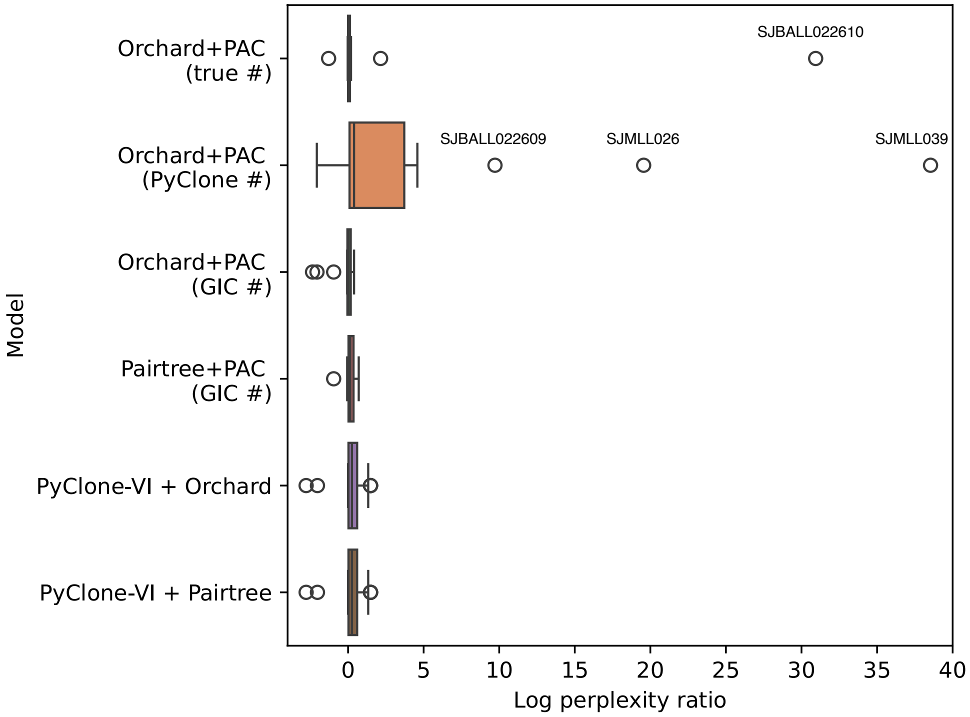

Fig N. The box plots illustrate the distribution of log perplexity ratios for trees inferred by phylogeny-aware clustering compared to clone trees reconstructed using clusters output by PyClone-VI across 14 B-ALL data sets from [17]. The log perplexity ratio is calculated relative to $F^{(\mathrm{MAP})}$ fitted to the expert-derived tree for each data set. Overall, the phylogeny-aware clustering algorithm using the GIC for model selection, "Orchard+PAC (GIC #)", performed the best.

- "Orchard+PAC (expert #)": phylogeny-aware clustering applied to Orchard's best reconstructed tree; manually selected clone tree with the same number of clones as the expert-derived tree.
- "Orchard+PAC (PyClone-VI #)": phylogeny-aware clustering applied to Orchard's best reconstructed tree; manually selected clone tree with the same number of clones as recovered by PyClone-VI.
- "Orchard+PAC (GIC #)": phylogeny-aware clustering applied to Orchard's best reconstructed tree; selected clone tree using GIC.
- "Pairtree+PAC (GIC #)": phylogeny-aware clustering applied to Pairtree's best reconstructed tree; selected clone tree using GIC.
- "PyClone-VI+Orchard": Clone tree reconstructed by Orchard using the clones recovered by PyClone-VI.
- "PyClone-VI+Pairtree": Clone tree reconstructed by Pairtree using the clones recovered by PyClone-VI.

To ensure a fair comparison among trees, we manually selected trees of the same size from the phylogeny-aware clustering output to compare against the expert-derived trees and the clone trees reconstructed using PyClone-VI's clusters. We only used PyClone-VI in this comparison because it performed the best among all VAF-based mutation clustering methods in our experiments.

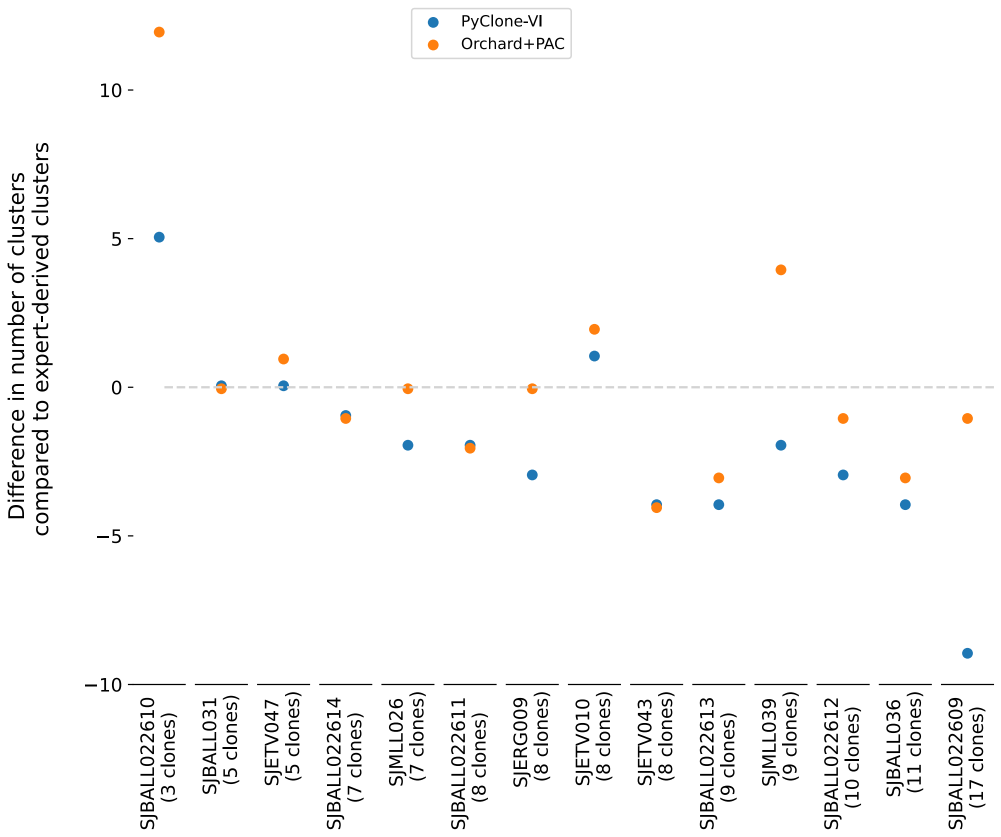

Fig O. Difference in the number of clones inferred by "Orchard+PAC (GIC #)" versus PyClone-VI [13] for the 14 B-ALL data sets. The number of clones inferred by each method is relative to the number of expert-derived clones. Each column shows the results for a single B-ALL data set. The horizontal gray dotted line represents no difference in the number of clusters inferred by a method compared to the expert-derived number. A point that lies far above or below this gray dotted line means that a method inferred a number of clone that's very different from the expert-derived number.

Fig N shows the distributions of log perplexity ratios for the clone trees reconstructed by each method on the 14 B-ALL data sets. The log perplexity ratio is computed in relation to $F^{(\mathrm{MAP})}$ for each of the expert-derived clone trees. Overall, "Orchard+PAC (GIC #)" generally found the best clone trees of any method. It's important to note that this method greatly overestimated the number of clones for SJBALL022610 compared to the expert-derived count. However, for all other data sets, the estimated number of clones was, on average, closer to the expert-derived count compared to PyClone-VI (see Fig O). One notable finding is that "Orchard+PAC (PyClone-VI #)" yielded several poor reconstructions when selecting the clone tree with the same number of clones as PyClone-VI's output. There are three B-ALL data sets identified as outliers for this method in Fig N that range from about +10-40 bits above

the baseline. We believe this finding is largely due to PyClone-VI inferring far fewer clones on the B-ALL data sets compared to the experts. For 10/14 B-ALLs PyClone-VI infers on average 3.4 fewer clones per data set compared to the expert-derived counts. In contrast to ”Orchard+PAC (PyClone-VI #)”, ”Orchard+PAC (expert #)” performs very well, with the only dramatic outlier being SJBALL022610. These findings suggest that, for all B-ALLs besides SJBALL022610, the phylogeny-aware clustering algorithm aligns more closely with the number of clones derived by experts compared to the numbers output by PyClone-VI. This is further supported by the results for “Orchard+PAC (GIC #)”, which selects a clone tree that, on average, differs by ±2 clones compared to the expert count, whereas PyClone-VI differs by ±2.5 clones (see Fig O). Overall, this experiment supports that our proposed method of inferring clones from a mutation tree can yield better clone trees compared to the classical approach of reconstructing clone trees using clusters output by a VAF-based clustering method.

A7 Tables

	Orchard (k=1)	Orchard (k=10)	Pairtree	CALDER
SJBALL022609	**0.016674**	0.016822	0.016706	2.617256
SJBALL022610	**0.000924**	*0.000924	*0.000924	1.648541
SJBALL022611	**0.000777**	0.000800	0.000791	0.444308
SJBALL022612	**0.003345**	0.003367	0.003348	-
SJBALL022613	**0.010827**	*0.010827	*0.010827	1.052539
SJBALL022614	**0.006357**	*0.006357	*0.006357	1.350474
SJBALL031	-0.000357	-0.000357	**-0.000386**	0.604383
SJBALL036	**0.034083**	0.054480	*0.034083	0.905851
SJERG009	**0.009929**	*0.009929	*0.009929	-
SJETV010	0.093638	**0.083516**	*0.083516	1.556607
SJETV043	**0.009944**	0.010110	0.010023	1.578067
SJETV047	**0.021099**	*0.021099	*0.021099	1.538149
SJMLL026	0.002358	0.002553	**0.002446**	1.201738
SJMLL039	*0.012682	*0.012682	**0.012682**	1.438552

Table A. Log perplexity ratio for each method on the 14 B-ALL clone tree reconstruction problems. Bold designates lowest (best) log perplexity ratio. A “*” represents a difference of less than 1e-6 compared to the best performing method. For display purposes, we do not show the full precision of the scores, so for some of the data sets, multiple methods may appear to have equal scores. Orchard (k=1) has a lower log perplexity ratio compared to Pairtree on 10/14 data sets.

	Orchard+PAC	Pairtree+PAC	PyClone-VI	VIBER	SciClone
SJBALL022609	0.806	**0.960**	0.503	0.626	0.121
SJBALL022610	0.273	0.356	0.730	**0.984**	0.019
SJBALL022611	**0.966**	0.825	**0.966**	**0.966**	0.857
SJBALL022612	**0.893**	0.891	0.891	0.888	0.742
SJBALL022613	0.919	0.904	**0.920**	0.914	0.696
SJBALL022614	**0.971**	**0.971**	**0.971**	0.855	0.709
SJBALL031	**1.0**	**1.0**	**1.0**	0.946	0.321
SJBALL036	**0.860**	**0.860**	0.829	0.815	0.167
SJERG009	**0.920**	**0.920**	0.875	**0.875**	0.0
SJETV010	0.497	0.428	0.440	0.469	**0.647**
SJETV043	**0.810**	**0.810**	**0.810**	**0.810**	0.619
SJETV047	**1.0**	**1.0**	**1.0**	0.977	0.0
SJMLL026	**1.0**	**1.0**	0.930	0.892	0.0
SJMLL039	0.927	0.927	**0.960**	0.907	0.014

Table B. Adjusted Rand Index (ARI) for each mutation clustering method on the 14 B-ALL data sets. Bold designates highest (best) ARI. If more than one method achieved the best ARI on a data set, all corresponding methods are bolded. "Orchard+PAC" and "Pairtree+PAC" refer to the phylogeny-aware clustering method applied to mutation trees constructed by Orchard and Pairtree, respectively.

Data set	Clusters
SJBALL022609	17
SJBALL022610	3
SJBALL022611	8
SJBALL022612	10
SJBALL022613	9
SJBALL022614	7
SJBALL031	5
SJBALL036	11
SJERG009	8
SJETV010	11
SJETV043	8
SJETV047	5
SJMLL026	7
SJMLL039	9

Table C. The number of clusters derived by experts in [17] for the 14 B-ALL data sets. The data can be found here: https://github.com/morrislab/pairtree-experiments

	Simulated data set size (mutations)											
	10		30		50		100		150		200	
	mean	*std*	*mean*	*std*	*mean*	*std*	*mean*	*std*	*mean*	*std*	*mean*	*std*
CALDER	< 1	< 1	99	188	468	201	NA	NA	NA	NA	NA	NA
Pairtree	1	< 1	6	1	11	3	46	28	90	28	229	38
Orchard (k=1)	< 1	< 1	< 1	1	< 1	< 1	6	6	18	12	34	17
Orchard (k=10)	< 1	< 1	1	1	4	2	60	63	198	134	374	182

Table D. Table of wall clock run time means and standard deviations (rounded to the nearest minute) for each method on each problem size for the simulated cancer reconstructions show in Figure 3. NA denotes a problem size where a method did not produce a reconstruction. < 1 represents that the mean or standard deviation of run times for a method on a problem size was less than a minute.

References

1. Wintersinger JA, Dobson SM, Kulman E, Stein LD, Dick JE, Morris Q. Reconstructing Complex Cancer Evolutionary Histories from Multiple Bulk DNA Samples Using Pairtree. Blood Cancer Discovery. 2022;3(3):208–219. doi:10.1158/2643-3230.BCD-21-0092.

2. Kulman E, Wintersinger J, Morris Q. Reconstructing cancer phylogenies using Pairtree, a clone tree reconstruction algorithm. STAR Protocols. 2022;3(4):101706. doi:10.1016/j.xpro.2022.101706.

3. Tarabichi M, Salcedo A, Deshwar AG, Ni Leathlobhair M, Wintersinger J, Wedge DC, et al. A practical guide to cancer subclonal reconstruction from DNA sequencing. Nature Methods. 2021;18(2):144–155. doi:10.1038/s41592-020-01013-2.

4. Jiao W, Vembu S, Deshwar AG, Stein L, Morris Q. Inferring clonal evolution of tumors from single nucleotide somatic mutations. BMC Bioinformatics. 2014;15(1):35. doi:10.1186/1471-2105-15-35.

5. El-Kebir M, Oesper L, Acheson-Field H, Raphael BJ. Reconstruction of clonal trees and tumor composition from multi-sample sequencing data. Bioinformatics. 2015;31(12):i62–i70. doi:10.1093/bioinformatics/btv261.

6. Kool W, van Hoof H, Welling M. Stochastic Beams and Where to Find Them: The Gumbel-Top-k Trick for Sampling Sequences Without Replacement; 2019. Available from: http://arxiv.org/abs/1903.06059.

7. Kool W, Hoof Hv, Welling M. Ancestral Gumbel-Top-k Sampling for Sampling Without Replacement. Journal of Machine Learning Research. 2020;21(47):1–36.

8. Jia B, Ray S, Safavi S, Bento J. Efficient Projection onto the Perfect Phylogeny Model. In: Advances in Neural Information Processing Systems. vol. 31. Curran Associates, Inc.; 2018.Available from: https://proceedings.neurips.cc/paper/2018/hash/d198bd736a97e7cecfdf8f4f2027ef80-Abstract.html.

9. Myers MA, Satas G, Raphael BJ. CALDER: Inferring Phylogenetic Trees from Longitudinal Tumor Samples. Cell Systems. 2019;8(6):514–522.e5. doi:10.1016/j.cels.2019.05.010.

10. Müllner D. Modern hierarchical, agglomerative clustering algorithms; 2011. Available from: http://arxiv.org/abs/1109.2378.

11. Neath AA, Cavanaugh JE. The Bayesian information criterion: background, derivation, and applications. WIREs Computational Statistics. 2012;4(2):199–203. doi:10.1002/wics.199.

12. Kim Y, Kwon S, Choi H. Consistent Model Selection Criteria on High Dimensions;.

13. Gillis S, Roth A. PyClone-VI: scalable inference of clonal population structures using whole genome data. BMC Bioinformatics. 2020;21(1):571. doi:10.1186/s12859-020-03919-2.

14. Caravagna G, Heide T, Williams MJ, Zapata L, Nichol D, Chkhaidze K, et al. Subclonal reconstruction of tumors by using machine learning and population genetics. Nature Genetics. 2020;52(9):898–907. doi:10.1038/s41588-020-0675-5.

15. Miller CA, White BS, Dees ND, Griffith M, Welch JS, Griffith OL, et al. SciClone: Inferring Clonal Architecture and Tracking the Spatial and Temporal Patterns of Tumor Evolution. PLOS Computational Biology. 2014;10(8):e1003665. doi:10.1371/journal.pcbi.1003665.

16. Santos JM, Embrechts M. On the Use of the Adjusted Rand Index as a Metric for Evaluating Supervised Classification. In: Alippi C, Polycarpou M, Panayiotou C, Ellinas G, editors. Artificial Neural Networks – ICANN 2009. Lecture Notes in Computer Science. Berlin, Heidelberg: Springer; 2009. p. 175–184.

17. Dobson SM, García-Prat L, Vanner RJ, Wintersinger J, Waanders E, Gu Z, et al. Relapse-Fated Latent Diagnosis Subclones in Acute B Lineage Leukemia Are Drug Tolerant and Possess Distinct Metabolic Programs. Cancer Discovery. 2020;10(4):568–587. doi:10.1158/2159-8290.CD-19-1059.
